# Exploring COVID‐19 education to support vaccine confidence amongst the general adult population with special considerations for healthcare and long‐term care staff: A scoping review

**DOI:** 10.1002/cl2.1352

**Published:** 2023-08-13

**Authors:** Maya Murmann, Anna Cooper Reed, Mary Scott, Justin Presseau, Carrie Heer, Kathryn May, Amy Ramzy, Chau N. Huynh, Becky Skidmore, Vivian Welch, Julian Little, Kumanan Wilson, Melissa Brouwers, Amy T. Hsu

**Affiliations:** ^1^ Bruyère Research Institute Bruyère Ottawa Ontario Canada; ^2^ Institute of Health Policy, Management and Evaluation University of Toronto Toronto Ontario Canada; ^3^ School of Epidemiology and Public Health University of Ottawa Ottawa Ontario Canada; ^4^ Ottawa Hospital Research Institute The Ottawa Hospital Ottawa Ontario Canada; ^5^ Civic Campus The Ottawa Hospital Ottawa Ontario Canada; ^6^ Department of Family Medicine University of Ottawa Ottawa Ontario Canada; ^7^ Department of Medicine University of Ottawa Ottawa Ontario Canada

## Abstract

**Background:**

Despite the demonstrated efficacy of approved COVID‐19 vaccines, high levels of hesitancy were observed in the first few months of the COVID‐19 vaccines' rollout. Factors contributing to vaccine hesitancy are well‐described in the literature. Among the various strategies for promoting vaccine confidence, educational interventions provide a foundationally and widely implemented set of approaches for supporting individuals in their vaccine decisions. However, the evidence around the measurable impact of various educational strategies to improve vaccine confidence is limited. We conducted a scoping review with the aim of exploring and characterizing educational interventions delivered during the pandemic to support COVID‐19 vaccine confidence in adults.

**Methods:**

We developed a search strategy with a medical information scientist and searched five databases, including Ovid MEDLINE and Web of Science, as well as grey literature. We considered all study designs and reports. Interventions delivered to children or adolescents, interventions on non‐COVID‐19 vaccines, as well as national or mass vaccination campaigns without documented interaction(s) between facilitator(s) and a specific audience were excluded. Articles were independently screened by three reviewers. After screening 4602 titles and abstracts and 174 full‐text articles across two rounds of searches, 22 articles met our inclusion criteria. Ten additional studies were identified through hand searching. Data from included studies were charted and results were described narratively.

**Results:**

We included 32 studies and synthesized their educational delivery structure, participants (i.e., facilitators and priority audience), and content. Formal, group‐based presentations were the most common type of educational intervention in the included studies (75%). A third of studies (34%) used multiple strategies, with many formal group‐based presentations being coupled with additional individual‐based interventions (29%). Given the novelty of the COVID‐19 vaccines and the unique current context, studies reported personalized conversations, question periods, and addressing misinformation as important components of the educational approaches reviewed.

**Conclusions:**

Various educational interventions were delivered during the COVID‐19 pandemic, with many initiatives involving multifaceted interventions utilizing both formal and informal approaches that leveraged community (cultural, religious) partnerships when developing and facilitating COVID‐19 vaccine education. Train‐the‐trainer approaches with recognized community members could be of value as trust and personal connections were identified as strong enablers throughout the review.

## 1 PLAIN LANGUAGE SUMMARY

### 1.1 Educational interventions for Covid‐19 vaccine confidence frequently involved group and individual‐based strategies

Many educational interventions were introduced to adult populations during the COVID‐19 pandemic, to help build their vaccine knowledge and confidence. These initiatives often involved the use of both formal (e.g., presentations) and informal (e.g., one‐on‐one conversations) approaches. Community partnerships were often leveraged to develop and facilitate COVID‐19 vaccine education.

### 1.2 What is this review about?

There were high levels of hesitancy towards the COVID‐19 vaccines when they were first introduced, despite scientific evidence showing their effectiveness in reducing morbidity and mortality from COVID‐19. Education about the vaccines was one of the key tools commonly used by public health professionals and healthcare providers to support individuals in their vaccine decisions.

**What is the aim of this review?**

This scoping review identifies and describes educational interventions delivered during the pandemic to support COVID‐19 vaccine confidence in adults. This review also highlights lessons learned that can be applied to support vaccine confidence in adults and priority populations for vaccinations, such as healthcare workers.

### 1.3 What studies are included?

The authors included all studies that described an educational intervention on COVID‐19 vaccines provided to adult populations, regardless of geography. The interventions were required to include an active interaction between an education facilitator and an audience member. For example, informational pamphlets about the Covid‐19 vaccines were excluded.

The authors found 32 studies from seven countries, published between February 2021 and February 2022. There were two rounds of database and grey literature searches.

### 1.4 What are the main findings of this review?

Group‐based educational interventions were used more frequently than individual‐based approaches. Formal presentations were the most common type of intervention. Other group‐based interventions included community events and small group discussions.

Individual‐based interventions included phone calls, in‐person consultations and door‐to‐door communication. Many interventions involved multiple strategies. Often, formal group‐based presentations were coupled with additional individual‐based interventions, such as one‐on‐one conversations either in‐person or on the phone.

Given the novelty of the COVID‐19 vaccines, personalized conversations, question periods, and other opportunities to address misinformation were important components of educational interventions. Healthcare professionals provided the education in almost all studies. Many of the studies also leveraged community leaders who were familiar to the audience to support the education. The studies were diverse in their priority populations.

### 1.5 What do the findings of this review mean?

This review identified many lessons that may be relevant to future vaccine education. For example, personal connections and trust between educator and audience is an important theme. Utilizing recognized community leaders could therefore be an important strategy.

Also, working with the audience to understand their preferences, such as how the education should be provided (i.e., format and language) and their information needs (i.e., common concerns) helps to ensure relevant and effective interventions.

### 1.6 How up‐to‐date is this review?

The review authors searched for studies published from the onset of the COVID‐19 pandemic to February 2022.

## 2 INTRODUCTION

Before the COVID‐19 pandemic, vaccine hesitancy was identified by the World Health Organization (WHO) as one of the ten threats to global health (WHO, 2021) as rates of vaccine‐preventable diseases, such as measles, have risen over recent years (Goldstein et al., 2020; Robert et al., 2019; WHO Regional Office for Africa, 2022). Vaccine hesitancy refers to the refusal or delay in acceptance of a vaccine despite the vaccine availability (WHO, 2021)—a critical concern throughout the COVID‐19 pandemic. Most countries prioritized the vaccination of healthcare workers (HCWs), especially those working in long‐term care (LTC), to be among the first to receive a COVID‐19 vaccine. Despite being at high risk for COVID‐19 (Dooling & Chamberland, 2020; Gharpure et al., 2021) and the risk of transmitting the virus to vulnerable patient populations, vaccine uptake among workers in the health and LTC sectors varied. Before the vaccine rollout, the proportion of HCWs around the world inclined to be vaccinated against COVID‐19 ranged from 28% to 82% (Research, Analysis, and Evaluation Branch Ministry of Health, 2021). In the United States, nearly half (48%) of frontline HCWs had not received a single dose of the vaccines against COVID‐19 by March 2021 (Research, Analysis, and Evaluation Branch Ministry of Health, 2021). High vaccine uptake is vital in high‐risk settings, such as the health and LTC sectors, to potentially protect vulnerable persons and to protect the workers themselves. As such, strategies to support vaccine confidence are imperative to address gaps in vaccine coverage and control COVID‐19 transmission.

Drivers of vaccine hesitancy, towards COVID‐19 and other vaccines, have been comprehensively studied and well‐described (European Centre for Disease Prevention and Control, 2015a). With respect to COVID‐19 vaccines, concerns around adverse side effects, safety, insufficient testing and efficacy were among the leading drivers of vaccine hesitancy. A variety of individual and group characteristics have also been identified as barriers to COVID‐19 vaccine acceptance, including being female (Biswas et al., 2021; Research, Analysis, and Evaluation Branch Ministry of Health, 2021), younger (Biswas et al., 2021; Research, Analysis, and Evaluation Branch Ministry of Health, 2021; Toth‐Manikowski et al., 2022), having lower education (Biswas et al., 2021; Toth‐Manikowski et al., 2022), working in rural settings (Research, Analysis, and Evaluation Branch Ministry of Health, 2021) and identifying as Black (Research, Analysis, and Evaluation Branch Ministry of Health, 2021; Toth‐Manikowski et al., 2022) or Latinx (Research, Analysis, and Evaluation Branch Ministry of Health, 2021). Despite our understanding of why people, including HCWs, might be vaccine‐hesitant, a significant knowledge gap exists with respect to the approaches and interventions that have been introduced to improve vaccine confidence. Few strategies to increase vaccine confidence have been rigorously and formally evaluated (Jarrett et al., 2015). Instead, most interventions rely on assumption‐based approaches (Jarrett et al., 2015).

Uptake of the primary series of COVID‐19 vaccination have increased over time within the health and LTC sectors (Razzaghi et al., 2022), with 88% of LTC staff in the United States having completed the primary series by September 2022 (Chidambaram & Burns, 2022; Drew, 2022; Karaivanov et al., 2022; Mills & Rüttenauer, 2022; Walkowiak et al., 2021). Emerging evidence attributes this rise in vaccination rates, including among HCWs and LTC staff, in part to the introduction of vaccination mandates (Kaiser Family Foundation, 2022). Vaccination mandates were found to be effective before the COVID‐19 pandemic for childhood vaccines, although less evidence is available for their efficacy on adult populations beyond healthcare settings (Mello et al., 2022; Schumacher et al., 2021). Mandates are a commonly used intervention, globally, and often employed by decision‐makers including governments, employers, as well as educational institutions for population‐level protection against transmissible diseases (Mello et al., 2022). However, this approach has important implications for health equity, trust in institutions (both scientific and government) (Bardosh et al., 2022), and on individual psychological resistance and reactance (Castillo et al., 2022). Key concerns of compulsory vaccination among HCWs include the erosion of trust, particularly among marginalized groups, and exacerbation of staff shortages (Woolf et al., 2022). Moreover, in most cases, mandates have not been extended to include COVID‐19 booster doses and uptake of boosters has remained low with just over half (51%) of LTC staff in the United States having received at least one booster by September 2022 (Chidambaram & Burns, 2022). As such, leveraging non‐coercive and trust‐building strategies, like education, will be important to establish and maintain trust, support vaccine confidence and ultimately increase vaccination coverage, including future booster dosages (Ontario COVID‐19 Science Advisory Table, 2021). However, poorly designed education that is complex and inaccessible may contribute to mistrust and challenge vaccine confidence (Yigit et al., 2021). To promote vaccine confidence, educational interventions must be designed in a manner that is conducive to the needs of the priority population.

Research on vaccine uptake among HCWs has been predominantly focused on the seasonal flu vaccine (Dini et al., 2018; European Centre for Disease Prevention and Control, 2015b; Hussain et al., 2018; Manuel et al., 2002; Oguz, 2019; Pereira et al., 2018; Schmid et al., 2017). Although some strategies may be transferable to COVID‐19 vaccines, COVID‐19 vaccine hesitancy is unique in many regards; most notably, the context of a global pandemic, partisan politics, the speed by which the vaccines were developed, and the use of novel mRNA technology. We, therefore, sought to identify and describe COVID‐19‐specific educational interventions that have been introduced to improve vaccine confidence, paying particular attention to those directed at the healthcare workforce.

## 3 OBJECTIVES

The primary objective of this scoping review was to identify and describe educational interventions that have been introduced to support COVID‐19 vaccine confidence in adults globally. The following research questions were explored:

1. What educational interventions have been developed to encourage COVID‐19 vaccine uptake and support vaccine confidence amongst the general adult population during the COVID‐19 pandemic?
2. What are the characteristics of these interventions?
3. What characteristics, if any, could be applied to educational interventions directed at the health and LTC workforce, given they are a prioritized group for vaccinations and the unique human resource considerations and challenges within these sectors?

## 4 METHODS

### 4.1 Protocol and registration

A scoping review methodology was used as it facilitates an exploratory approach to mapping an emerging concept using a range of published and unpublished literature. We followed the Joanna Briggs Institute Evidence Synthesis Reporting Guide for Protocols and the Preferred Reporting Items for Systematic reviews and Meta‐Analyses extension for Scoping Reviews (PRISMA‐ScR) statement (Tricco et al., 2018) for the reporting of our methods and results. The protocol was peer‐reviewed, and published on Campbell Systematic Reviews (Reed et al., 2022).

### 4.2 Information sources and search

Two rounds of literature searches were conducted, each of which contained a database and grey literature search. The first database and grey literature searches were done in July 2021 and August 2021, respectively, to capture early evidence shortly following the approval and initial rollout of the COVID‐19 vaccines. The second database and grey literature searches were preformed in February 2022 to capture mature evidence more than 1 year post‐COVID‐19 vaccine approval. This allowed us to compare the evidence across two time periods and identify ways in which the approaches to vaccine education evolved over time.

We developed and piloted search strategies through an iterative process in consultation with an experienced medical information scientist. The MEDLINE strategy was peer‐reviewed by another senior information scientist before execution using the Peer Review of Electronic Search Strategies (PRESS) Checklist. We used the Ovid platform to search the following databases: Ovid MEDLINE®, including Epub Ahead of Print, In‐Process & Other Non‐Indexed Citations, Embase Classic+Embase, and APA PsycInfo.

We also carried out a grey literature search of selected sites listed in CADTH's Grey matters (CADTH, 2021). Additionally, we searched the following COVID‐19‐specific resources: Cochrane COVID‐19 Study Register (Cochrane COVID‐19 Study Register, 2022), Covid‐END (McMaster Health Forum, 2022), L‐OVE (Epistemonikos, 2022), LTCcovid.org (International Long‐Term Care Policy Network, 2022), unCoVer (unCoVer, 2022), the COVID‐19 resources from ClinicalTrials.gov (ClinicalTrials.gov, U.S. National Library of Medicine, 2022) and the WHO COVID‐19 Database (WHO, 2022). We also searched CINAHL (EBSCO Information Services) and Web of Science (‘Core Collection’). Lastly, a manual search of the reference lists of included studies was also conducted to identify any literature that was not captured in the original search. Refer to Supporting Information: eFiles 1a and 1b for full details of the databases, search strategy, and key terms.

### 4.3 Eligibility criteria

All study designs and papers were considered to ensure our review captured developing evidence, as well as anecdotal experiences of COVID‐19 vaccine education delivery. Preprints and publications in languages other than English were excluded. No date restrictions were used. While the intent was to identify strategies for healthcare settings, this review included COVID‐19 vaccine educational interventions delivered to other populations in a variety of contexts. This was done for two reasons: first, our preliminary searches that focused specifically on COVID‐19 vaccine educational interventions prioritizing healthcare workers yielded very few results; second, we suspected that broadening to other populations and contexts would unveil important lessons learned and strategies that are relevant the health and LTC sectors. As COVID‐19 is a global pandemic, we did not restrict publications to a specific geography. However, we excluded national or mass vaccination campaigns as our focus was on educational interventions involving engaged learning at the time of delivery (i.e., interactions between the facilitator and the participants) and that can be reproduced at an institutional level within hospitals or LTC facilities. This review considered only interventions delivered to adult populations and excluded any education developed and delivered to either children or adolescent populations. Finally, reports or studies focused on non‐COVID‐19 related vaccines were also excluded.

We included studies and reports describing informally and formally delivered education on the COVID‐19 vaccines. Education was defined as an informative action, interaction, or intervention. Formal education is often characterized as education that is didactic, guided or systematic (Pereira et al., 2018). It generally is introduced through a rigid curriculum and delivered in ‘formal institutions’ (Pereira et al., 2018) and may include, workshops or seminars. Informal education is described by Spaan and colleagues as any unstructured or opportunistic interactions that take place outside of formal training and are ‘in the control of the learner’ (Dini et al., 2018). Studies on educational resources or tools with no clearly described delivery and interaction between facilitator and participant(s) (i.e., passive education) were excluded. These include generic emails, information handouts, or online e‐learning requirements, for example.

### 4.4 Selection of sources of evidence

Titles and abstracts of all citations were screened independently by two of five reviewers (ACR, MM, AR, MS, CNH) using Covidence (Veritas Health Innovation, 2022). Studies were selected according to pre‐defined criteria, as stated above. Full texts of potentially eligible papers for inclusion were retrieved, uploaded into Covidence and screened independently by two reviewers. Any screening conflicts that arose were discussed amongst the research team. Data extraction from included studies was performed by one reviewer and validated by a second reviewer.

### 4.5 Data items, chart processing and synthesis of results

**Figure 1:**
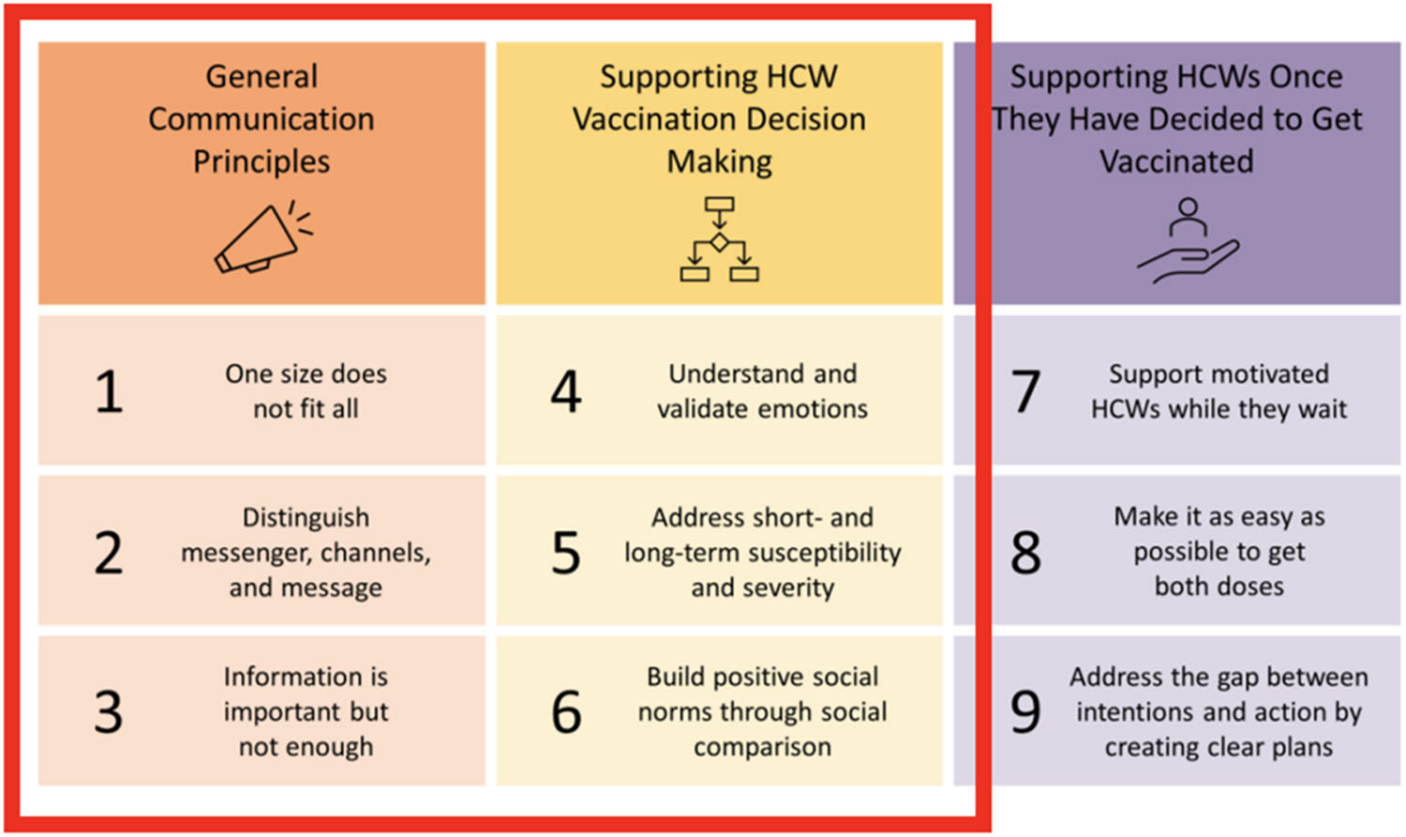
Nine principles for supporting vaccination confidence and uptake in health care workers. We focused on Principles 1 through 6 as these were relevant to the decision to be vaccinated and within the scope of this study. *Source*: Presseau et al. (2021).

Data analysis was informed by the Behavioural Science Principles for Supporting COVID‐19 Vaccine Confidence and Uptake Among Ontario Health Care Workers (Figure 1), a scientific brief produced by the Ontario COVID‐19 Science Advisory Table on key evidence‐based behaviour change strategies that can be employed to support vaccine confidence and uptake in HCWs (Presseau et al., 2021). Of the nine proposed principles, only six were directly applicable to our educational strategy‐focused research questions and preceded an individual's decision to accept the vaccine. Guided by these principles, we developed a data charting tool (Supporting Information: eFile 2), which was refined and revised as appropriate following the extraction of a small number of studies. Furthermore, the presentation of results was informed by Principle #2, which suggests distinguishing between the messenger, channel and message. The results are presented according to the delivery format (i.e., channel), type of participants (including the facilitators (i.e., messenger) and audience), as well as content (i.e., message). The remaining principles were applied, primarily within the content section, as a form of deductive coding of the data.

When possible, data was displayed in a tabular format and accompanied by a narrative summary of how the data relate to the objectives and research questions. Emphasis was placed on findings that are directly relevant to the healthcare and LTC workforces. Comparisons were made between studies included in the first (i.e., early evidence) and second (i.e., mature evidence) literature searches.

## 5 RESULTS

### 5.1 Selection of sources of evidence

This review included a total of 32 studies captured during two searches performed on July 21, 2021 and February 11, 2022. The first round yielded 13 studies while the second round yielded 19 studies. All studies in Round 2 were independent and not related to a study captured in Round 1.

During the first round, we screened the titles and abstracts of 4601 articles. The peer‐reviewed database search yielded 1917 potentially relevant studies without any duplicates. Following title and abstract screening, 72 full text articles were reviewed, of which 65 were excluded and 7 were included. The grey literature search retrieved 2684 studies after one duplicate article was removed. Following title and abstract screening, 45 full text articles were reviewed, of which we excluded 41 and included 4. Two additional studies were retrieved from hand searching.

**Figure 2:**
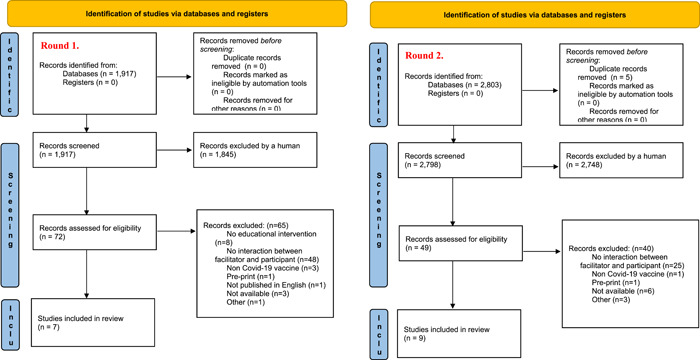
PRISMA flowchart. *Source*: Page et al. (2021).

During the second round, we screened the titles and abstracts of 4693 articles. The peer‐reviewed database search yielded 2798 potentially relevant studies after five duplicates were removed. Following title and abstract screening, 49 full text articles were reviewed, of which 40 were excluded and 9 were included. The grey literature search yielded 1895 studies after four duplicate articles were removed. Following title and abstract screening, eight full text articles were reviewed, six of which were excluded while two were included. Eight additional studies were retrieved from hand searching. See Figure 2 for the PRISMA chart.

### 5.2 Characteristics of sources of evidence

**Figure 3:**
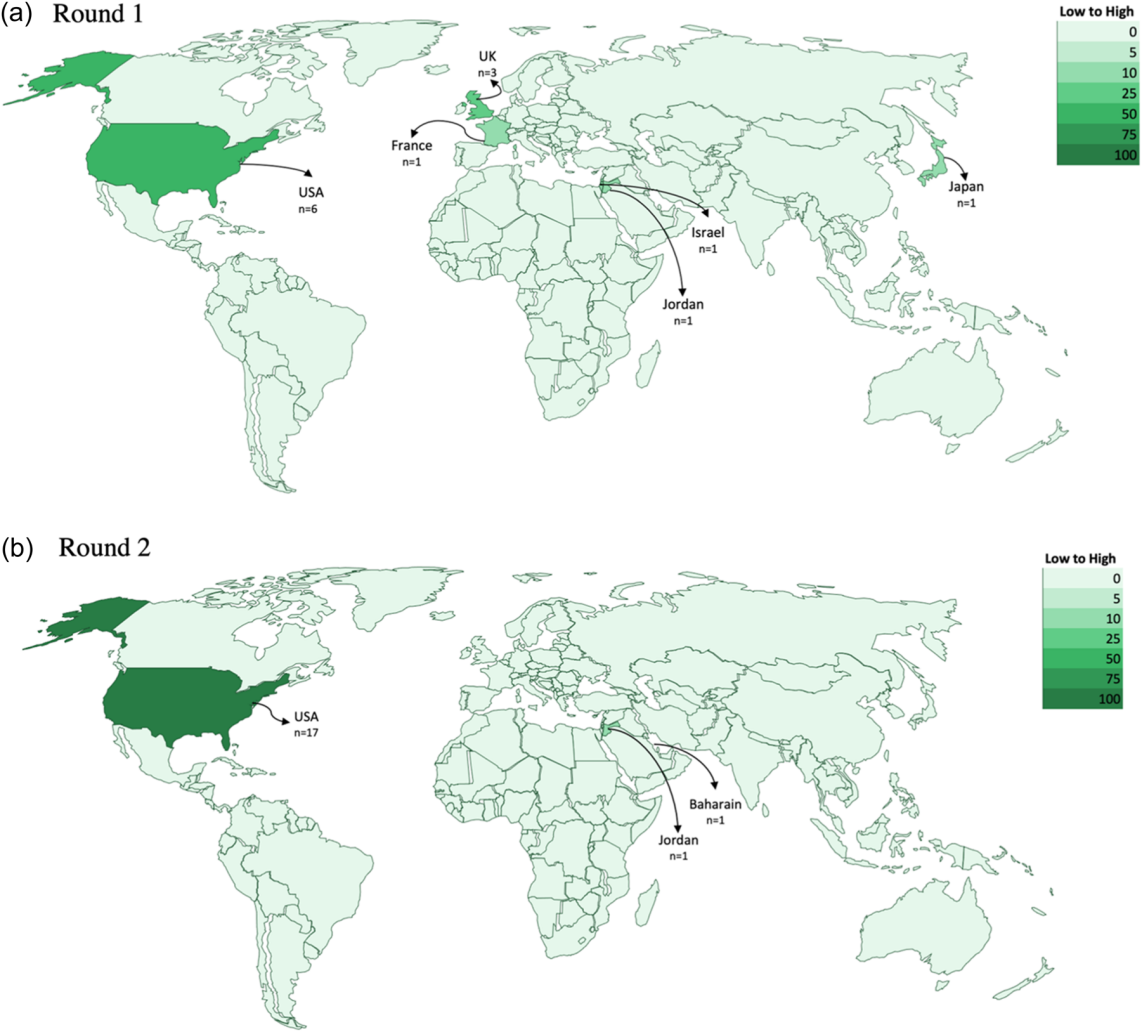
Geographic distribution of included studies on educational interventions for COVID‐19 vaccine uptake Published Between February 2021 and February 2022.

Included studies were from seven countries (Figure 3) and consisted of 14 journal articles, one research letter, two journal news articles, three reports, eight news articles and one of each of the following: commentary, letter to the editor, perspective and practice note. There was consistency between studies with respect to the objectives of the educational interventions introduced. Namely, they were primarily designed to provide information to increase knowledge (Gakuba et al., 2021; Kelkar et al., 2021; National Institute for Health and Care Excellence [NICE], 2021); to allow individuals to make informed choices (Berry et al., 2021; Moberly, 2021); to encourage COVID‐19 vaccine uptake and acceptance (Gakuba et al., 2021; Ginder‐Vogel, 2021; Marquez et al., 2021; Moberly, 2021; NHS England, 2021; Quinn & Andrasik, 2021; Serper et al., 2021; Spelman et al., 2022a; Takamatsu et al., 2021; Traynor, 2021); to address COVID‐19 misinformation (Bouchard, 2021; Hopper, 2021; Tesfaye, 2022); to reduce hesitancy (Abou Leila et al., 2021; Gakuba et al., 2021; Li et al., 2022; NHS England, 2021; Traynor, 2021); to boost confidence (Gakuba et al., 2021; NHS England, 2021; Traynor, 2021); and to support communities disproportionately impacted by COVID‐19 (AuYoung et al., 2022; Marquez et al., 2021; Wagner et al., 2022; WCAX, 2021).

### 5.3 Synthesis of results

#### 5.3.1 Delivery format of the educational interventions (channel)

##### Group‐based versus individual‐based interventions

**Figure 4:**
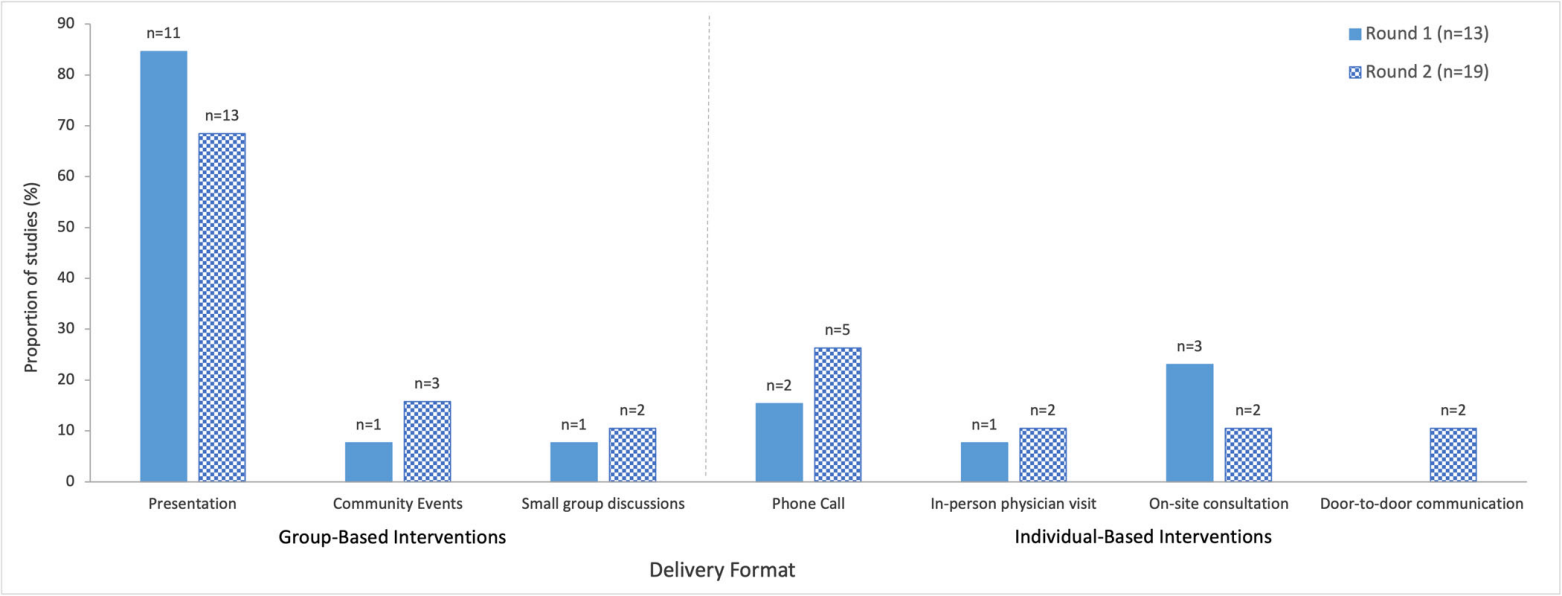
Delivery format of educational interventions examined in included studies on educational interventions for COVID‐19 vaccine uptake published February 2021 and February 2022. Categories were not mutually exclusive.

**Figure 5:**
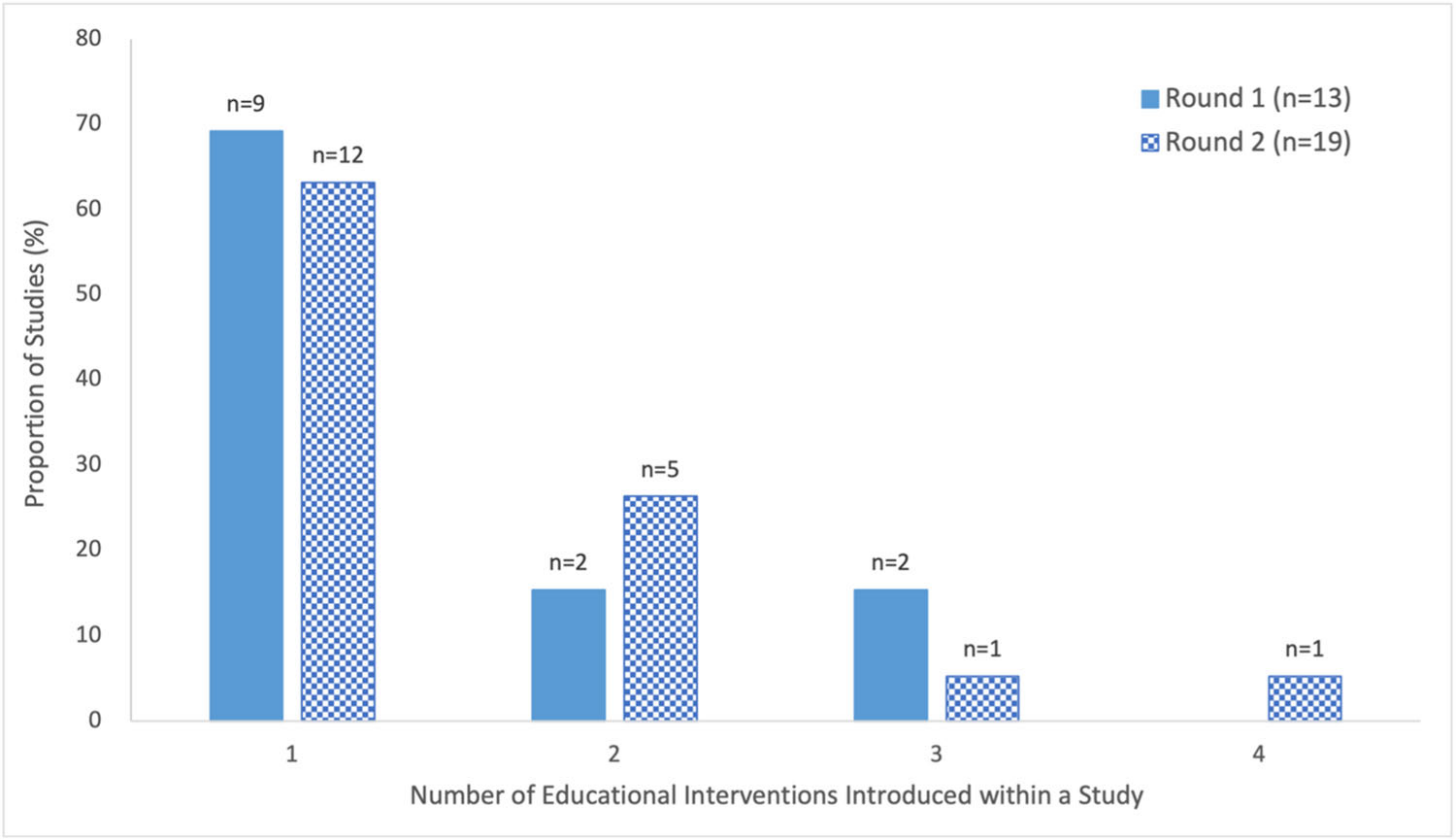
Number of educational interventions introduced in included studies on educational interventions for COVID‐19 vaccine uptake published Between February 2021 and February 2022.

Group‐based educational interventions were used more frequently across both rounds of literature searches (Round 1: 92%, Round 2: 79%) relative to individual‐based interventions (Round 1: 38%, Round 2: 53%) (Figure 4, Supporting Information: eTable 1). Group‐based interventions included presentations, community events (AuYoung et al., 2022; Marquez et al., 2021; Moberly, 2021; Scott et al., 2021), and small group discussions (AuYoung et al., 2022; Feifer et al., 2021; Garcia, 2021), while individual‐based interventions included phone calls (AuYoung et al., 2022; Moberly, 2021; NHS England, 2021; Rosario, 2021; Serper et al., 2021; Spelman et al., 2022a; Wiley, 2021), in‐person physician visits, in‐person and on‐site counselling or consultations (i.e., within the priority populations' local setting, such as a nursing home or military base) (Feifer et al., 2021; Marquez et al., 2021; Rosario, 2021; Takamatsu et al., 2021; Talmy et al., 2021) as well as door‐to‐door communication (Ginder‐Vogel, 2021; Marquez et al., 2021). Most studies utilized a single intervention (Round 1: 69%, Round 2: 63%), however, approximately a third of studies introduced at least two educational interventions (Round 1: 30%, Round 2: 36%) (Figure 5, Supporting Information: eTable 2).

**Figure 6:**
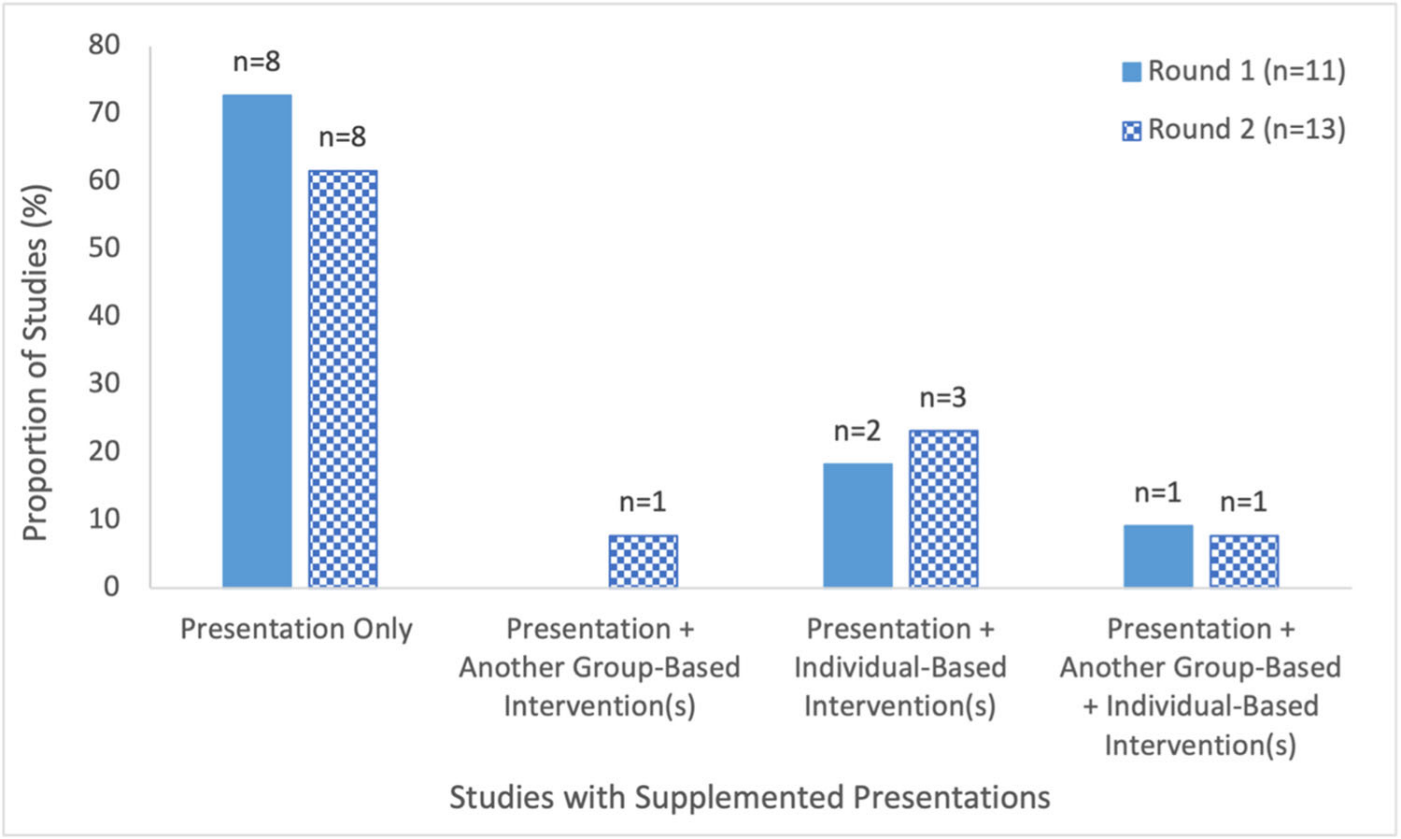
Number of studies that supplemented presentations with additional interventions in included studies on educational interventions for COVID‐19 vaccine uptake published Between February 2021 and February 2022.

Presentations were the most commonly employed format for delivering educational intervention across both rounds (Round 1: 85%, Round 2: 68%) (Figure 4, Supporting Information: eTable 1). Presentations included, for example, webinars (Kelkar et al., 2021; NICE, 2021; Peteet et al., 2021; Traynor, 2021), coaching sessions (Abdel‐Qader et al. 2021, 2022), town halls (AuYoung et al., 2022; Berry et al., 2021; Garcia, 2021; Ginder‐Vogel, 2021; Hopper, 2021; Wagner et al., 2022), lectures (Abou Leila et al., 2021; Talmy et al., 2021), educational sessions (Spelman et al., 2022a; Takamatsu et al., 2021), information sessions (Gakuba et al., 2021; WCAX, 2021; Wiley, 2021), ‘Ask a Doc’ sessions (Bouchard, 2021; Feifer et al., 2021), train‐the‐trainer sessions (Quinn & Andrasik, 2021), and Q&A sessions (Garcia, 2021; Tesfaye, 2022). Virtual presentations whereby participants were able to ask questions, allowed for meaningful active dialogue and bidirectional communication between the speakers and audience, as noted (AuYoung et al., 2022). Some studies offered presentations on a repeated basis, although only one study specifically mentioned a rationale for doing so, which was to ‘enabl[e] [nursing home] employees to log in from home and invite members of their family to participate [throughout the day or night]’ (Feifer et al., 2021). The majority of presentations were conducted virtually (Round 1: 64%, Round 2: 69%) (Supporting Information: eTable 1). Roughly a quarter (27%) and one‐third (31%) of studies in the first and second round, respectively, supplemented a presentation with an additional individual‐based intervention (Figure 6).

An increase in the introduction of individual‐based interventions was observed in Round 2, with over half (53%) of studies employing at least one type of one‐on‐one intervention, the most common of which was a phone call (26%) (Supporting Information: eTable 1). In Round 1, phone calls typically took the form of a healthcare professional communicating directly with their patients. This was also observed in Round 2, however, education through phone calls also took place in the form of information hotlines (AuYoung et al., 2022; Wiley, 2021), a format not observed in the earlier round. Door‐to‐door communication was also observed only in the second round. Individual‐based one‐on‐one conversations, whether through phone calls or other modalities, may lend more opportunities for facilitators to understand and validate the emotions of their audience (Figure 1, principle #4) and address specific concerns related to COVID‐19's short and long‐term susceptibility and severity (Figure 1, principle #5).

Several studies also included passive educational approaches in addition to the interactive interventions, such as information leaflets, email announcements, e‐learning requirements and social media posts, which did not fulfill our inclusion criteria and were therefore not included.

##### Virtual versus in‐person

**Figure 7:**
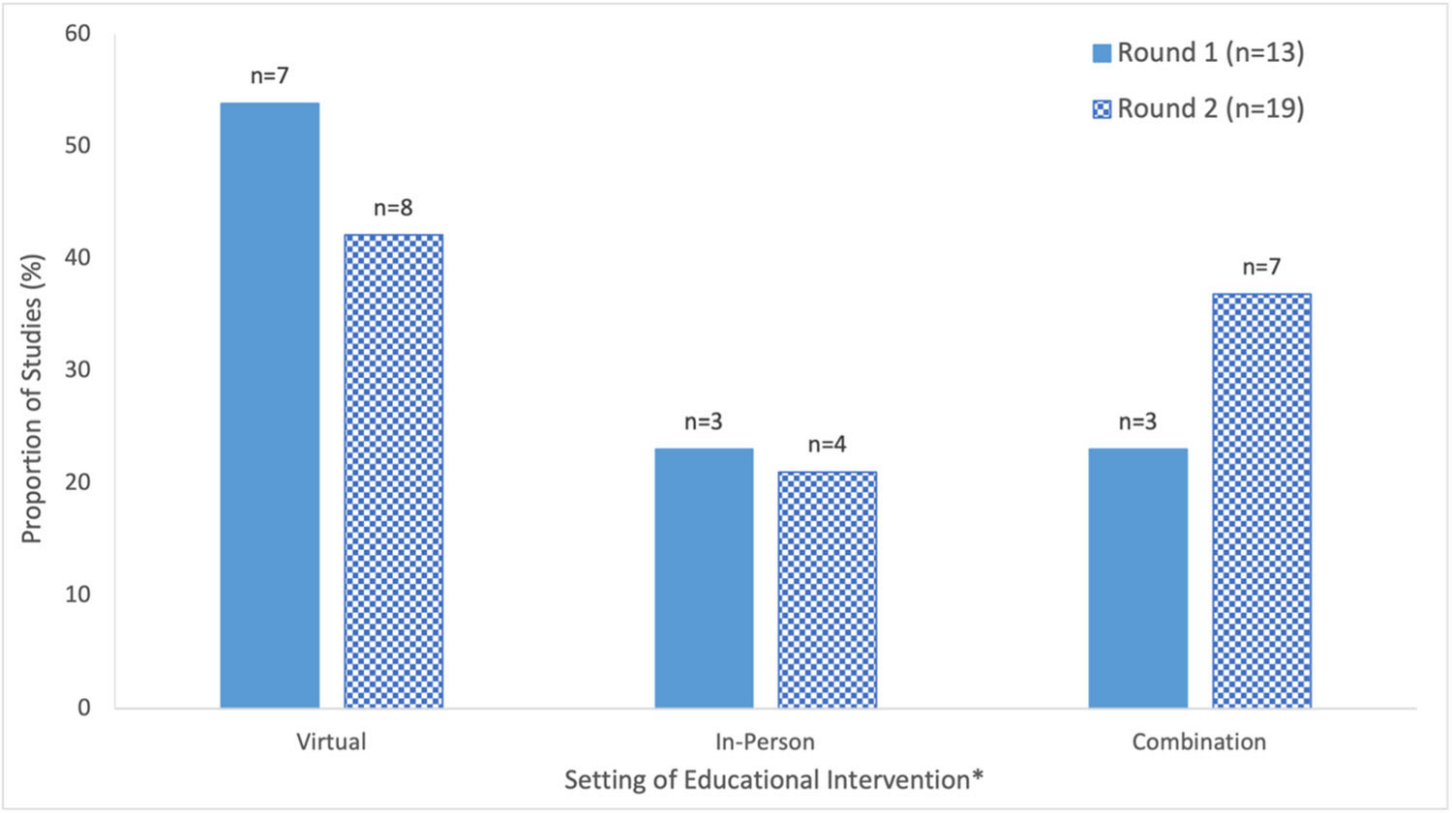
Setting (virtual or in‐person) of interventions in included studies on educational interventions for COVID‐19 vaccine uptake published between February 2021 and February 2022. One study from Round 1 (Takamatsu et al., 2021) and two studies from Round 2 (Abou Leila et al., 2021; Spelman et al., 2022a) included two educational interventions, one of which was unclear whether it was conducted in‐person or virtually.

A decrease in the proportion of studies employing exclusively virtual education was observed between the first and second round (Round 1: 54%, Round 2: 42%), with a greater proportion of studies in Round 2 leveraging a combination of in‐person and virtual educational interventions compared to the first round (Round 1: 23%, Round 2: 37%) (Figure 7). This may be attributable to changes in the COVID‐19 requirements and vaccination status. One justification for utilizing a virtual platform was that it allowed the educational material to be more accessible (i.e., can be recorded, made available online for public access, and thus reviewed offline by the audience) (Abdel‐Qader et al., 2022). However, in‐person education was used in one study as opposed to pre‐recorded sessions to promote interactions with participants and improve participation rates (Gakuba et al., 2021). In‐person education was also noted to be valuable for individuals seeking a personal connection and those without access to the internet (AuYoung et al., 2022).

##### Train‐the‐trainer

A train‐the‐trainer model was employed in one‐quarter of studies in Round 1 (24%) (Abdel‐Qader et al., 2021; Berry et al., 2021; Quinn & Andrasik, 2021) and one‐third of studies in Round 2 (37%) (Abdel‐Qader et al., 2022; Abou Leila et al., 2021; AuYoung et al., 2022; Ginder‐Vogel, 2021; Katzman et al., 2022; Marquez et al., 2021; Spelman et al., 2022a). This model was frequently leveraged to increase the human resource capacity for vaccine education. It typically involved equipping facilitators with the necessary knowledge and skills required to be able to provide effective and tailored COVID‐19 vaccine education (Abdel‐Qader et al., 2022), a finding aligned with the principle that ‘one size does not fit all’ (Figure 1, principle #1). This included, for instance, intensive and interactive training on health coaching principles, verbal and non‐verbal communication skills (Abdel‐Qader et al., 2021; Abou Leila et al., 2021), models of behaviour change (Abdel‐Qader et al., 2021) and COVID‐19 information (Abou Leila et al., 2021). Most often, this training was provided to healthcare professionals, including pharmacists (Abdel‐Qader et al. 2021, 2022; Ginder‐Vogel, 2021), physicians (Abdel‐Qader et al., 2022; Abou Leila et al., 2021; Quinn & Andrasik, 2021; Spelman et al., 2022a), opinion leaders characterized as ‘outspoken staff member that other individuals may listen to, regardless of whether the staff member planned to receive the COVID‐19 vaccine’ (Berry et al., 2021), and community health teams (Marquez et al., 2021; Quinn & Andrasik, 2021). In one study, changes to the School of Pharmacy curriculum were made to equip faculty with the knowledge and skills necessary to respectfully work with diverse populations (Ginder‐Vogel, 2021). This included the introduction of a ‘lecture about the history of racism in medical research’ (Ginder‐Vogel, 2021), which may allow facilitators to more effectively understand and validate emotions of various priority audiences (Figure 1, principle #4). In two studies, community members received training to establish community partnerships allowing for the continued dissemination of COVID‐19 education (Marquez et al., 2021; Quinn & Andrasik, 2021). For instance, clients at vaccination sites received education from site staff regarding how to motivate unvaccinated family friends, tips on handling challenging conversations, and engaged in role‐play of hypothetical scenarios to boost confidence (Marquez et al., 2021). The objective of this model was to empower vaccinated clients to become ‘vaccine ambassadors’ in recognition of the important impact of vaccinated people in influencing the beliefs, attitudes, knowledge and uptake of COVID‐19 vaccines within their social network (Marquez et al., 2021). In doing so, positive social norms may be established more broadly (Figure 1, principle #6).

#### 5.3.2 Educational participants: Priority audience and facilitators (messenger)

##### Audience

**Figure 8:**
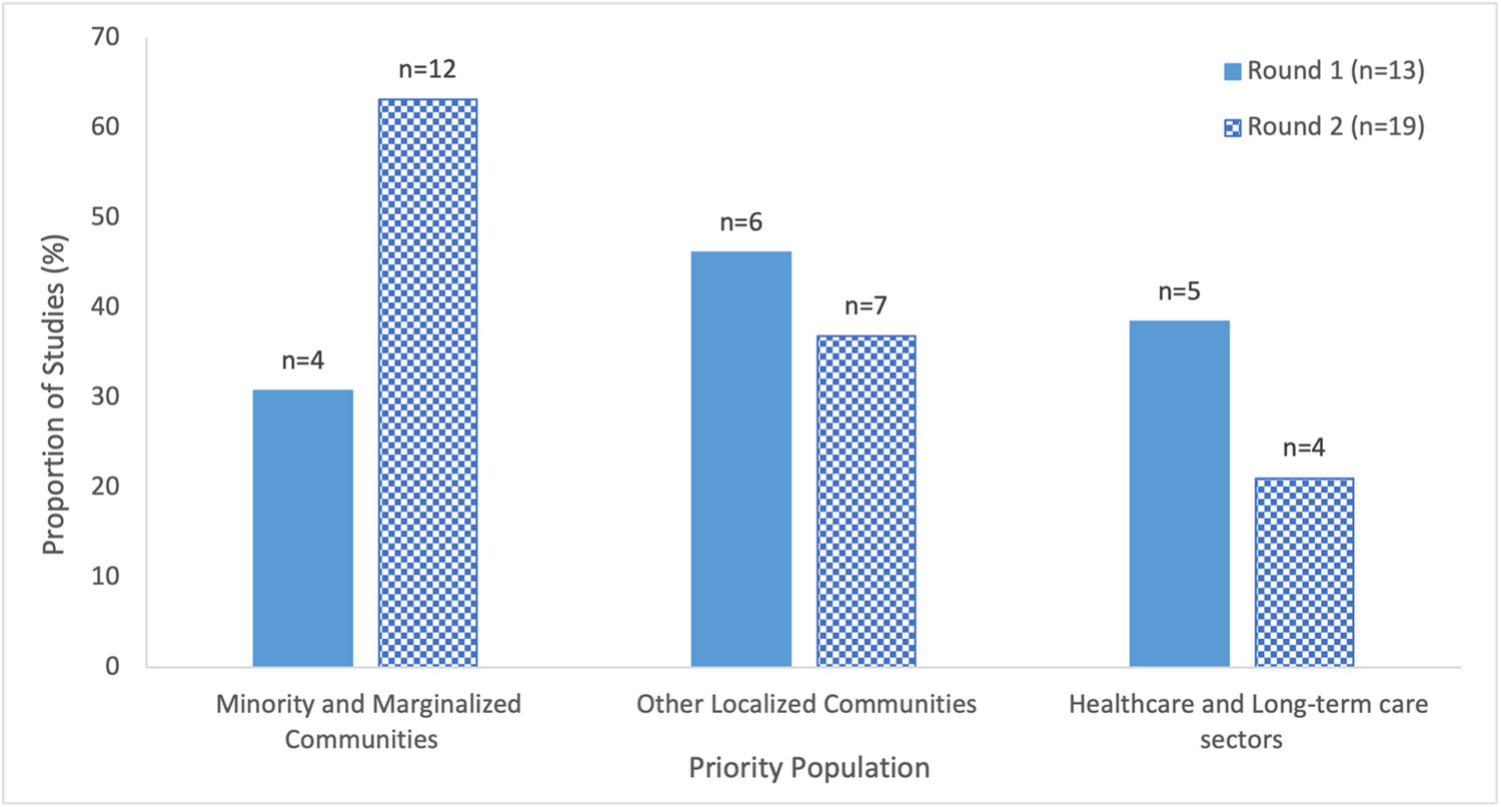
Priority population of interventions in included studies on educational interventions for COVID‐19 vaccine uptake published between February 2021 and February 2022. Categories were not mutually exclusive.

The included studies were diverse in their priority populations (Figure 8, Supporting Information: eTable 3). Education was provided to vaccine‐hesitant individuals or those who had not received the vaccine within a localized community in almost half of studies in Round 1 (46%) and over a third of studies in Round 2 (37%). Localized communities included, for example, patient groups (Abou Leila et al., 2021; Hirshberg et al., 2021; Kelkar et al., 2021; Moberly, 2021; NHS England, 2021; Serper et al., 2021; Spelman et al., 2022a), individuals within a pharmacy network (Abdel‐Qader et al., 2021; Traynor, 2021), and soldiers (Li et al., 2022; Talmy et al., 2021). In one study, educational outreach was staggered with an initial phase directed at the highest‐risk individuals within a specified community followed by a larger‐scale rollout in the second phase (Spelman et al., 2022a). An increase in the proportion of studies whereby education was directed towards minority and marginalized communities was observed between Round 1 (31%) and Round 2 (63%). Lastly, those in the healthcare or LTC sectors were the priority population in over a third (38%) and one‐fifth (21%) of studies in Round 1 and 2, respectively. This included healthcare personnel in a tertiary‐care centre (Takamatsu et al., 2021), Intensive Care Unit staff (Gakuba et al., 2021), nursing home staff and their families (Feifer et al., 2021), physicians/clinicians (Abdel‐Qader et al., 2022; Katzman et al., 2022), pharmacists (Abdel‐Qader et al., 2022), community health workers (AuYoung et al., 2022), and public health practitioners (Katzman et al., 2022). No educational interventions were specifically directed towards LTC staff in Round 2.

##### Facilitators (messenger)

**Figure 9:**
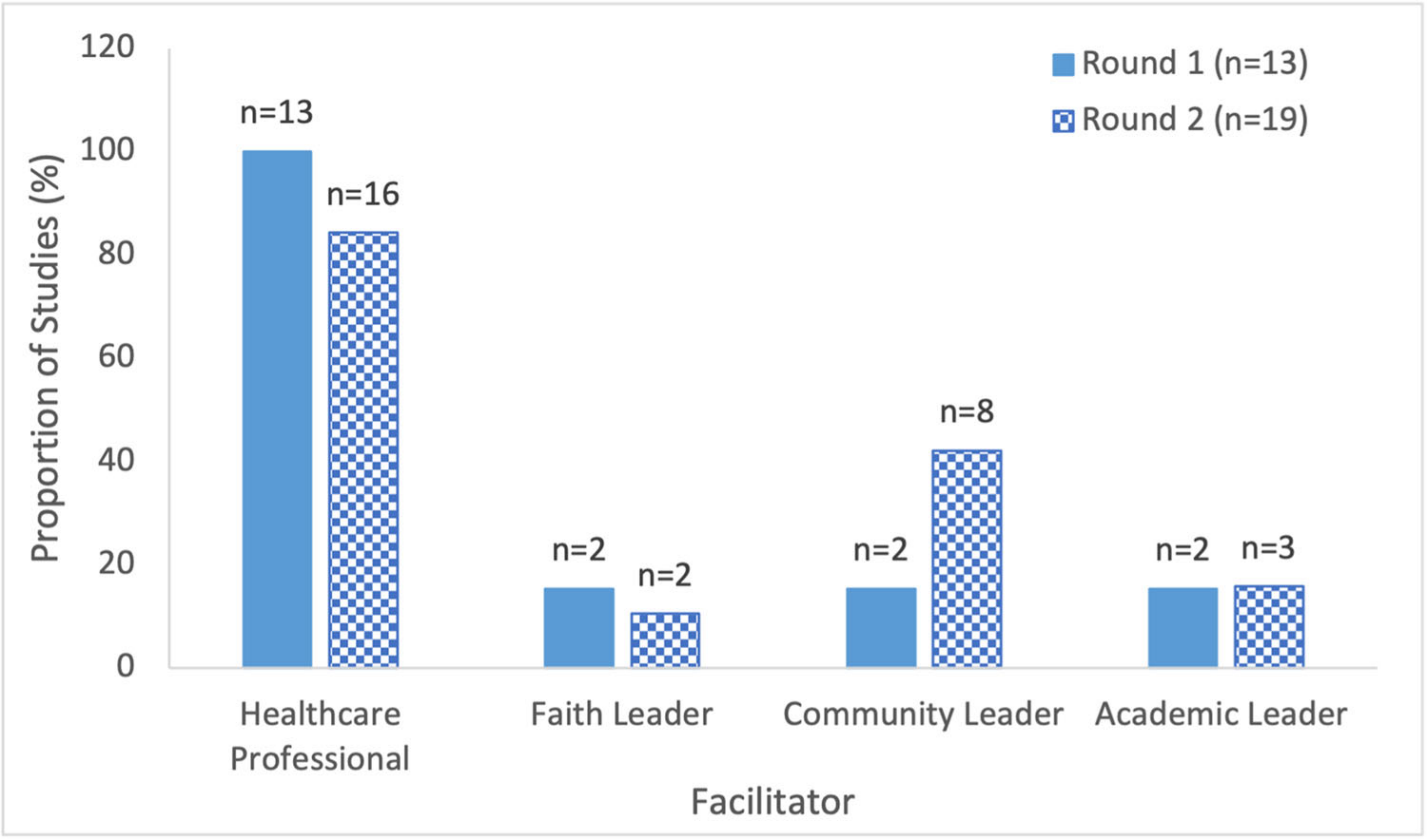
Type of interventions facilitators in included studies on educational interventions for COVID‐19 vaccine uptake published between February 2021 and February 2022. Categories were not mutually exclusive.

**Figure 10:**
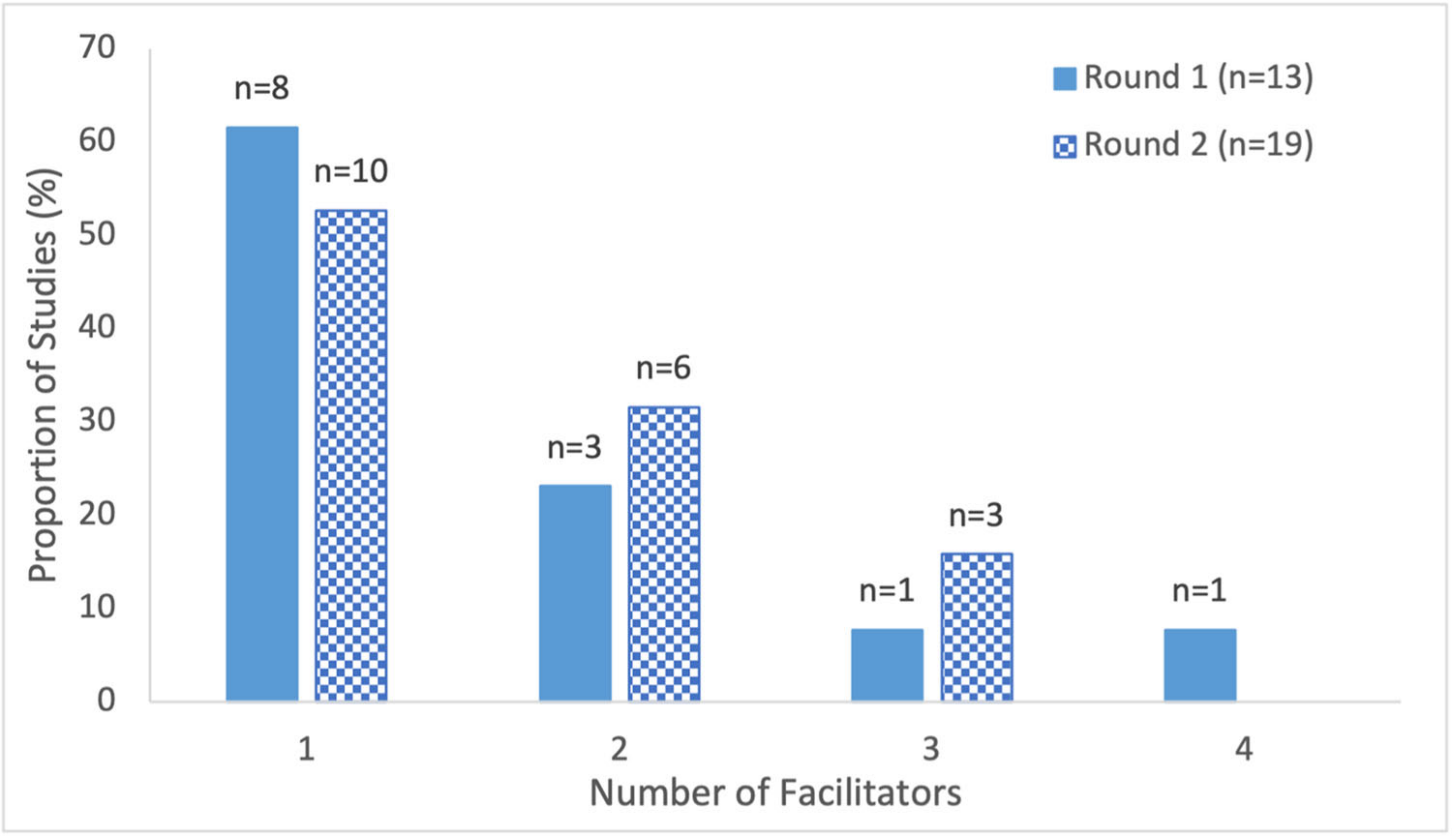
Number of intervention facilitators in included studies on educational interventions for COVID‐19 vaccine uptake published between February 2021 and February 2022.

**Figure 11:**
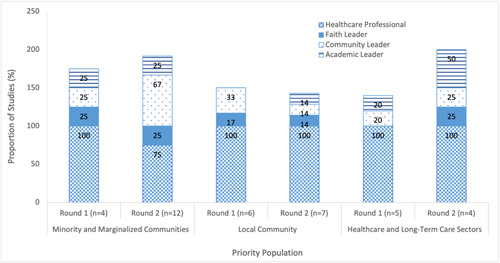
Facilitators stratified by priority population in included studies on educational interventions for COVID‐19 vaccine uptake published between February 2021 and February 2022.

All but three studies, all of which were from Round 2, involved a healthcare professional in the facilitation of the educational intervention (Supporting Information: eTable 4). Additional facilitators leveraged within the studies included community leaders, faith leaders and academic leaders (the vast majority of whom had medical expertise) (Figure 9). Examples of community leaders, who facilitated the education in almost half (42%) of studies in Round 2, include local peer influencers (Moberly, 2021), cancer patient advocates (Kelkar et al., 2021), a COVID‐19 trial recipient (NICE, 2021), cultural community health outreach programs (Peteet et al., 2021), community‐based organizations (Bouchard, 2021; Ginder‐Vogel, 2021; Marquez et al., 2021; Wagner et al., 2022; WCAX, 2021) and community advocates (AuYoung et al., 2022). A slight increase in collaboration between different types of facilitators was observed in Round 2, with the proportion of studies leveraging only a single facilitator decreasing from 62% to 53% between Rounds 1 and 2 (Figure 10 Supporting Information: eTable 5). Academic leaders were engaged more frequently among educational interventions directed towards healthcare workers while community leaders were leveraged in over two‐thirds of studies (67%) within which the priority population were minority and marginalized communities (Figure 11).

Several studies noted the important role of facilitators in establishing trust and that ‘a primary path toward trust and confidence is relationship building’ (Quinn & Andrasik, 2021). This was often achieved by leveraging a personal connection between the facilitator and audience; for example, the director of a pharmacy residency program, who is a facilitator from one study, stated ‘we know them, they know us—the pharmacists, the technicians, everybody involved in the process’ (Takamatsu et al., 2021). Health professionals, such as physicians and pharmacists, are considered by many as trustworthy sources of health information (Abdel‐Qader et al., 2022) and they played an important role in many educational interventions that were examined. Trust was also achieved through the involvement of facilitators who represented the audience ethnically, religiously, culturally (Berry et al., 2021; Bouchard, 2021; Feifer et al., 2021; Ginder‐Vogel, 2021; NHS England, 2021; NICE, 2021; Peteet et al., 2021), and linguistically (AuYoung et al., 2022; Feifer et al., 2021; Marquez et al., 2021; Peteet et al., 2021; WCAX, 2021; Wiley, 2021). The selection of facilitators who were considered trusted messengers was crucial for interventions directed towards minority communities, which have been disproportionately impacted by COVID‐19. As Quinn and Andrasik noted, reciprocal relationships between ‘Black, Indigenous, and People of Color (BIPOC) communities’ and medical/research institutions have been largely absent with underlying mistrust rooted in research abuses and governmental actions (Quinn & Andrasik, 2021). Accordingly, community‐engaged partnerships were leveraged in several studies in an effort to establish a foundation of trust (AuYoung et al., 2022; Wagner et al., 2022), inform content development and implementation of culturally‐sensitive strategies (Marquez et al., 2021), and maximize engagement (Wagner et al., 2022). For example, education was provided by bilingual or bicultural leaders familiar to the audience in one study, who were able to draw on and integrate cultural references within the education (AuYoung et al., 2022). Another study noted that this approach promoted a positive perception of the facilitators by participants, which may have influenced their motivation to absorb the information and subsequently change behaviour (Wagner et al., 2022). In fact, according to one study that leveraged a community‐academic partnership, recommendations from a trusted source was identified as the most important factor in visiting a vaccination centre among 20% of clients (Marquez et al., 2021).

Given the diversity of the LTC workforce, the inclusion of diverse facilitators was also highlighted in the two studies prioritizing this sector (Berry et al., 2021; Feifer et al., 2021), both from Round 1. For instance, in Berry et al., town halls were facilitated by moderators and geriatricians who ‘were racially and ethnically diverse’ in ‘hope to create a diverse cadre of trusted [skilled nursing facility] staff who would share accurate information with peers’ (Berry et al., 2021). Likewise, Feifer et al. (2021) utilized ‘Ask a Doc’ sessions introduced to nursing home staff and families where they included co‐panellists who were diverse in terms of ‘race, ethnicity, religion, cultural background, sexual orientation, age, disability status, and military service’ as well as linguistically to facilitate ‘culturally sensitive discussions’ (Feifer et al., 2021). Additionally, small group discussions and one‐on‐one conversations with staff were held by racially diverse nursing leaders (Feifer et al., 2021).

#### 5.3.3 Content of the educational interventions (message)

##### Addressing misinformation

Addressing misinformation was a common component of the educational interventions reviewed due to the significant degree of inaccurate information, largely transmitted through social media, surrounding the COVID‐19 vaccine. Dispelling fears and discussing circulating disinformation was a strategy explicitly identified in 13 studies (Round 1: 38% (Abdel‐Qader et al., 2021; NHS England, 2021; NICE, 2021; Talmy et al., 2021; Traynor, 2021), Round 2: 42% (Marquez et al., 2021; AuYoung et al., 2022; Bouchard, 2021; Garcia, 2021; Hopper, 2021; Li et al., 2022; Rosario, 2021; Tesfaye, 2022)). One study addressed misinformation due to evidence suggesting much of COVID‐19 vaccine hesitancy among the study population was associated with conspiracy beliefs (Abdel‐Qader et al., 2021). Three studies in Round 2 (16%) also dispelled inaccurate information regarding vaccine eligibility; for instance, clarifying immigration status does not impact eligibility (Bouchard, 2021; Garcia, 2021; Tesfaye, 2022).

##### Opportunities for questions

Another important element of many educational interventions reviewed was the opportunity for participants to ask questions. This allowed for the dissemination of targeted information to address specific concerns or questions participants may have had. In nearly all presentations (Round 1: 65% (Berry et al., 2021; Feifer et al., 2021; Gakuba et al., 2021; Peteet et al., 2021; Quinn & Andrasik, 2021; Talmy et al., 2021; Traynor, 2021), Round 2: 100% (Marquez et al., 2021); Abou Leila et al., 2021; AuYoung et al., 2022; Bouchard, 2021; Garcia, 2021; Hopper, 2021; Katzman et al., 2022; Li et al., 2022; Spelman et al., 2022a; Tesfaye, 2022; Wagner et al., 2022; WCAX, 2021; Wiley, 2021)), participants were given the opportunity to ask questions, often in the form of a Q&A session or panel. Presentation‐based interventions utilizing virtual platforms may provide participants with the option to ask questions anonymously (Li et al., 2022; Wagner et al., 2022) and in real‐time through chat functions that were integrated into the ongoing discussion (Wagner et al., 2022). This allowed for the interactions between participants and experts to be more dynamic (Bouchard, 2021). In one study, questions were collected before the intervention as part of the registration process and questions not addressed during the educational intervention were answered offline (Wagner et al., 2022). Individual‐based interventions also often allowed participants to consult the educator regarding any specific concerns privately providing an opportunity for the facilitator to understand and validate the emotions of the participants whilst providing vaccine education (Figure 1, principle #4). As reflected by one General Practitioner (GP) in a study who provided phone calls to their patients: ‘it's about giving people the time and the permission to ask the questions’ (Moberly, 2021). Similarly, the director of a pharmacy residency program referenced above emphasized the importance of ‘connect[ing] with patients, understand[ing] their concerns’ (Traynor, 2021).

##### Personal stories

The sharing of a facilitators' personal decision‐making process in receiving the COVID‐19 vaccine and eliminating their own hesitancy was an identified strategy to boost vaccine confidence in four studies (13%) (Abdel‐Qader et al., 2022; Berry et al., 2021; Ginder‐Vogel, 2021; Peteet et al., 2021; Traynor, 2021), and serves as examples of building positive norms through social comparison (Figure 1, principle #6). The townhall format, in one study, allowed peers to share their own personal stories related to the COVID‐19 vaccine with one another, which was identified as an ‘added benefit’ of this format (Berry et al., 2021).

##### Content development

Studies consistently emphasized the value and importance of providing education tailored to the priority population and that ‘one size does not fit all’ (Figure 1, principle #1). In addition to tailoring the education to the unique concerns of an individual through the opportunity to ask questions, the content of educational interventions was also tailored linguistically (AuYoung et al., 2022; Marquez et al., 2021; Rosario, 2021; Tesfaye, 2022; Wagner et al., 2022), culturally (Marquez et al., 2021; Wagner et al., 2022) and religiously (Abdel‐Qader et al., 2022) in several studies, particularly among those directed towards a minority community.

Uniquely, three studies performed an information‐gathering phase in advance of the formal education rollout to develop and disseminate content in an effective manner for the priority population. For instance, one study conducted focus groups inclusive of marginalized communities to develop tailored education that met the contextual and local preferences and needs of their communities (AuYoung et al., 2022). This led to the modification of terminology to address the implications of word choices on trust and the potential to inadvertently place blame on an individual. Specifically, the terms ‘vaccine confidence” or “vaccine deliberation” were used instead of “vaccine hesitancy,” while “herd immunity” was replaced with “community immunity” (AuYoung et al., 2022). Context‐specific education, in terms of the format, content and language was then developed. This is in line with the behavioural principle ‘information is important but not enough’, which encourages the reconsideration of terminology used (Figure 1, Principle 3). Another study conducted a five‐step formative phase over a 3‐month time period to gather information about the preference and characteristics of the priority community to develop appealing and engaging townhalls (Wagner et al., 2022). This included: drafting of educational materials, processes and assessments; conducting community consultations to gain feedback on the linguistic and cultural appropriateness of the material and to assess the appeal, relevance and accessibility of the education; revising the material accordingly; conducting practice town halls for another round of feedback; and lastly finalizing the materials, processes and assessments (Wagner et al., 2022). In another study, a recognition of the importance of authentic community engagement led to frequent discussions with community partners to identify community fears as a means to inform efforts to overcome them (Quinn & Andrasik, 2021).

Two studies provided a more standardized approach through the development of formal discussion tools to support facilitators providing the education. In the first study, a standardized discussion tool was designed to assist providers in counselling pregnant and breastfeeding patients (Hirshberg et al., 2021). The tool included answers to frequently asked questions as well as a scoring system to determine whether the patient should be vaccinated (BJC HealthCare & Washington University Physicians, 2021). In the second study (Spelman et al., 2022a), a scripted step‐wise note was developed by a primary care physician champion and vaccine acceptance subject matter expert following a review of relevant evidence‐based information and toolkits. The tool, called the COVID‐19 Prevention Telephone Note, consisted of discussion prompts, informed by motivational interviewing frameworks, for use by primary care patient–aligned care medical home teams (Spelman et al., 2022b).

Another study whereby education took the form of patient counselling by a physician, interviews with selected patients following their counselling sessions were used to provide regular performance feedback (Abou Leila et al., 2021).

## 6 DISCUSSION

### 6.1 Summary of evidence

Lack of COVID‐19 vaccine knowledge has been identified as a barrier to vaccine uptake among HCWs (Crawshaw et al., 2022). In fact, HCWs had an almost twofold greater odds of accepting a COVID‐19 vaccine if they had high COVID‐19 vaccine knowledge compared to those with low knowledge (Petravić et al., 2021) highlighting the importance of education. This scoping review therefore sought to summarize existing literature on educational interventions introduced to address COVID‐19 vaccine hesitancy to identify strategies that may be transferable to the healthcare and LTC workforces. To ensure the review was comprehensive, we included studies from any population across a wide range of sources. The findings from this review highlight the importance of tailoring education to the prioritized communities. As pandemic‐specific evidence grew, we found that more studies sought to engage with priority populations and utilized iterative methods to inform the design of educational intervention as well as content development. This was done to ensure that the educational interventions addressed the specific needs of its intended audience. Tailoring was also achieved in many studies through individual interactions, which allowed the facilitator to address the specific concerns and unique needs of the participant. Studies also demonstrated that educational efforts introduced to target COVID‐19 vaccine hesitancy and promote confidence strongly relied on the foundation of a trusting relationship between facilitators and the audience. Trust was frequently established by leveraging a facilitator who was familiar to the audience, including community leaders, community pharmacists, or physicians, as well as other healthcare professionals. This finding is supported by other existing literature on the positive effect of a trusted community member's endorsement on vaccination uptake (Marquez et al., 2021) for the influenza vaccine (Peterson et al., 2019), human papillomavirus vaccine (Gottvall et al., 2010) and childhood vaccines (Banerjee et al., 2010).

Hesitancy towards the COVID‐19 vaccine is complex and multilayered, and overcoming them will require a multipronged approach. Ensuring facilitators are equipped with the knowledge to respectfully work with diverse communities is critical to be able to relate with patients and establish trust (Ginder‐Vogel, 2021). As such, it may be of value for future educational programs to consider a train‐the‐trainer component, as was done in several of the included studies, before implementing an intervention to ensure facilitators themselves are adequately prepared to engage with the priority audience, utilize appropriate communication strategies, have an awareness of circulating disinformation and answers to frequently asked questions. While the training of educators will likely be time intensive (Omer et al., 2021), it will ensure educators are well‐equipped to support individuals who are vaccine‐hesitant. Moreover, train‐the‐trainer models can be used to establish a social network allowing for the rapid diffusion of information when the initial priority audience, now equipped with vaccine‐related knowledge, can act as a credible source of information to their peers (Marquez et al., 2021). This model is being used more frequently within public health to increase the reach and integration of interventions (Hunter et al., 2019; Marquez et al., 2021).

This review also found that formal, group‐based presentations were the most common type of educational intervention utilized to disseminate COVID‐19 vaccination education in the included studies across both rounds. Many studies, roughly one‐third in both rounds, supplemented presentations with an individual‐based intervention. Likewise, approximately a third of the studies utilized more than one educational intervention within a multi‐component strategy. Given the complexity of COVID‐19 vaccine hesitancy, the utilization of several educational interventions in parallel is likely required (Berry et al., 2022).

Given the novelty of the COVID‐19 vaccine as well as the unique context of the global vaccine rollout, many studies emphasized addressing misinformation, question periods and personal stories as important components of the educational approaches. These strategies allowed the facilitator to understand and validate the emotional responses of participants, build positive social norms, and address common COVID‐19 related questions.

### 6.2 Special considerations for HCWs and LTC staff

Although we found few studies targeting health care and LTC sectors initially, which prompted us to broaden our scope, we still saw that this was a priority population where initial educational interventions and research were applied. These findings can inform future educational interventions introduced within the health care and LTC settings, particularly in the context of an unexpected pandemic or outbreak. HCWs, particularly those who work in LTC, will likely remain a prioritized population for future vaccines given the health vulnerability of the population they support. However, any rollout of educational interventions in either of these sectors could be impacted by unique challenges which were, for the most part, not identified in our included studies as most were not focused on these sectors. These challenges could include operating on a 24‐h schedule, high staff turnover (Iida et al., 2021), understaffing (Stolee et al., 2005), high workloads with limited time to engage in education (Stolee et al., 2005), significant job‐related stress (Caspar et al., 2016), and limited resources (Caspar et al., 2016; Iida et al., 2021; Stolee et al., 2005), including physical spaces and equipment (Stolee et al., 2005). Specifically, within LTC homes, finding coverage and securing backfill for staff to participate in education is a noted challenge (Stolee et al., 2005).

It is evident then that administrators and educators need to be mindful of the development and rollout of education in these settings to boost confidence in COVID‐19 vaccines and beyond. Train‐the‐trainer offers a potential education model for the efficient diffusion of educational information considering the challenges related to staffing, timing and resources. Staff champions may be well‐positioned for this model as well, particularly since peer staff champions who reinforce the designated messaging have been found to be associated with higher levels of COVID‐19 vaccination among LTC staff (Berry et al., 2022). This is also supported by existing literature on the adoption of interventions and strategies in nursing homes. For example, in two studies that sought to improve advance care planning for nursing home patients through the introduction of a video‐based intervention, staff champions had a positive effect on the outcome of interest (Loomer et al., 2019; Palmer et al., 2019).

Additionally, multi‐component educational interventions that can be rolled out in phases and strengthen consistent messaging may likewise address the 24/7 shift‐work operational model and timing constraints. The benefits of this approach have been previously documented in one study that investigated the LTC facility‐level incentives, policies, activities and communication formats associated with higher COVID‐19 staff vaccination coverage (Berry et al., 2022). Specifically, the study found that LTC homes with medium to high COVID‐19 vaccination coverage were more likely to use a greater number of strategies compared to homes with low staff vaccination (Berry et al., 2022). This was similar to the findings in a systematic review of randomized controlled trials on interventions to improve the uptake of the influenza vaccine among HCWs; they found a positive association between multimodal education and vaccination coverage (Rashid et al., 2016). Moreover, given that the LTC workforce is predominately foreign‐born and female (OECD, 2020), ensuring education is facilitated by a trusted figure will likely be important to address any mistrust within this population. Facilitators should be a trusted member of the community, such as a religious or community leader (Bazan & Akgün, 2021; Long et al., 2021) who can understand and address personal concerns in a culturally safe and sensitive manner. Facilitators who are demographically and linguistically reflective of the population are likewise critical (Abdul‐Mutakabbir et al., 2021; Bazan & Akgün, 2021). Lastly, awareness of cultural considerations influencing vaccine beliefs will also be an important priority area for this workforce to ensure educational information is culturally tailored (Omer et al., 2021). To achieve this, educational interventions would likely benefit from an interactive process whereby facilitators engage with the priority population to identify their needs and preferences regarding the content, format, and language of the education to ensure effective, appropriate, and relevant messaging. Education should then be assessed and modified accordingly. This form of integrated knowledge translation, whereby knowledge users are involved in the research and development process, can increase knowledge use and uptake (McIsaac et al., 2018; Nguyen et al., 2020). Although the co‐development through the inclusion of participants, such as LTC staff, in educational development has also been found to foster a sense of ownership (Nolan et al., 2008), this practice has not frequently been employed among intervention studies within LTC facilities before the COVID‐19 pandemic (Caspar et al., 2016).

### 6.3 Limitations

Our study has several limitations. First, the wide breadth of the review across geographies, time and populations presented challenges for synthesizing this heterogeneous literature. Second, our last search was done in February 2022. Given the rapid and time‐sensitive nature of the COVID‐19 vaccination campaigns, we expect additional literature will be published and provide further clarity on this topic. As such, recent studies in this area are absent. Third, the ‘early’ versus ‘mature’ evidence classification was determined by publication date as opposed to the date the educational intervention was conducted. As such, educational interventions within studies considered ‘mature’ may have been conducted during similar time frames as studies captured in Round 1 and would therefore not be considered as more ‘mature’. However, as the pandemic evolved, public health communications pertaining to the need and benefits of the vaccines have also changed, in terms of their sense of urgency, and shifted from the perspective of eliminating risks at the population level to an individual level. Our review reflected these changes and we believe the evolution in vaccine education strategies was captured by our approach. Lastly, our review identified a limited number of studies in the health and LTC sectors, which were our target population at the outset of this study. We, therefore, had to draw from lessons that were derived from other contexts with few informed directly by the experiences of these sectors (Figure  11).

An important area for future work would be to investigate the effectiveness and impact of educational interventions on vaccine confidence. This review has identified common characteristics of educational approaches introduced across a variety of populations, thereby highlighting the components of educational interventions that would be important and interesting to examine. However, given multifaceted and complex interventions are challenging to evaluate, rigorous evaluation methods should be leveraged, such as a randomized control trial, to minimize potential bias that may arise from a pre‐post study design including susceptibility to the natural erosion of vaccine hesitancy over time. This review could also inform the design of a systematic review of COVID‐19 hesitancy in the general adult population, or a subset of the population based on the priority populations identified (e.g., healthcare workers, marginalized communities, etc.), with respect to the types of educational interventions that have been leveraged.

## 7 CONCLUSION

This scoping review provides a summary of emerging themes among educational interventions introduced to support COVID‐19 vaccine confidence which can inform future implementation research and practice aligned with the *behavioural principles* previously developed (Presseau et al., 2021). Our review suggests that educational interventions should consider leveraging community (patient, cultural, religious) partnerships when developing and facilitating COVID‐19 vaccine education. Equity‐focused and multifaceted strategies accounting for the ethical, diverse and inclusive considerations of the priority audience may be needed to increase COVID‐19 vaccination uptake. Train‐the‐trainer approaches with recognized community members could also be of value, as trust and personal connections were identified as an enabler throughout the review. While COVID‐19 vaccines have been mandated in some jurisdictions for health and LTC workforces, these findings could support endeavours aiming to encourage vaccine confidence or vaccination uptake, as well as contribute to any future need for boosters or new vaccines.

## CONTRIBUTIONS OF AUTHORS

Content: MM, ACR, MS, JP, CH, KM, AR, CNH, VW, JL, KW, MB, ATH

Systematic review methods: BS, VW

Statistical analysis: N/A

Information Retrieval: MM, ACR, MS, AR, CNH

## DECLARATIONS OF INTEREST

Vivian Welch is editor in chief and interim CEO of the Campbell Collaboration. She was not involved in the editorial process or decision to publish for this manuscript. No other conflicts of interest to declare.

## PLANS FOR UPDATING THIS REVIEW

None.

## DIFFERENCES BETWEEN PROTOCOL AND REVIEW

No methodological differences.

## SOURCES OF SUPPORT

**External Sources**: This work has been funded in part by the Canadian Institutes of Health Research (Funding reference no. GA3‐177726) and by the Public Health Agency of Canada (PHAC) through the COVID‐19 Immunity Task Force (CITF). Ce projet a été financé en partie par l'Agence de la santé publique du Canada (ASPC) par l'intermédiaire du Groupe de travail sur l'immunité face à la COVID‐19 (GTCI). The views expressed herein do not necessarily represent the views of the Public Health Agency of Canada.

**Internal Sources**: None.

## Supporting information

Supporting information.
