# Supplementary material for: Exploring COVID‐19 education to support vaccine confidence amongst the general adult population with special considerations for healthcare and long‐term care staff: A scoping review
Source: Campbell Syst Rev. 2023 Aug 13;19(3):e1352. doi: 10.1002/cl2.1352 (PMC10423318; doi:10.1002/cl2.1352)
Supplement: Supplementary file 1 — Supporting information. [file CL2-19-e1352-s001.docx]

# Supplementary Material

## eFile 1a. Medline Search Strategy

Vaccine Confidence

Final Strategies

2021 Jul 21

Ovid Multifile

Database: Ovid MEDLINE: Epub Ahead of Print, In-Process & Other Non-Indexed Citations, Ovid MEDLINE® Daily and Ovid MEDLINE® <1946-Present>, Embase Classic+Embase <1947 to 2021 July 20> , APA PsycInfo <1806 to July Week 2 2021>

Search Strategy:

--------------------------------------------------------------------------------

1 COVID-19/ (96621)

2 SARS-CoV-2/ (91732)

3 Coronavirus/ (14454)

4 Betacoronavirus/ (40876)

5 Coronavirus Infections/ (56750)

6 (COVID-19 or COVID19).tw,kf. (278693)

7 ((coronavirus* or corona virus*) and (hubei or wuhan or beijing or shanghai)).tw,kf. (10124)

8 (wuhan adj5 virus*).tw,kf. (518)

9 (2019-nCoV or 19nCoV or 2019nCoV).tw,kf. (3124)

10 (nCoV or n-CoV or "CoV 2" or CoV2).tw,kf. (99410)

11 (SARS-CoV-2 or SARS-CoV2 or SARSCoV-2 or SARSCoV2 or SARS2 or SARS-2 or severe acute respiratory syndrome coronavirus 2).tw,kf. (101222)

12 (2019-novel CoV or Sars-coronavirus2 or Sars-coronavirus-2 or SARS-like coronavirus* or ((novel or new or nouveau) adj2 (CoV or nCoV or covid or coronavirus* or corona virus or Pandemi*2)) or (coronavirus* and pneumonia)).tw,kf. (37641)

13 (novel coronavirus* or novel corona virus* or novel CoV).tw,kf. (19017)

14 ((coronavirus* or corona virus*) adj2 "2019").tw,kf. (62678)

15 ((coronavirus* or corona virus*) adj2 "19").tw,kf. (10097)

16 (coronavirus 2 or corona virus 2).tw,kf. (32349)

17 (OC43 or NL63 or 229E or HKU1 or HCoV* or Sars-coronavirus*).tw,kf. (7712)

18 COVID-19.rx,px,ox. or severe acute respiratory syndrome coronavirus 2.os. (7280)

19 (coronavirus* or corona virus*).ti. (45527)

20 ("B.1.1.7" or "B.1.351" or "B.1.617" or "B.1.617.2" or "B.1.427" or "B.1.429").tw,kf,rx,px,ox. (896)

21 ("P.1" and (Brazil* or variant?)).tw,kf,rx,px,ox. (3568)

22 ((alpha or beta or delta or gamma or lambda) adj3 variant?).tw,kf. (12128)

23 or/1-22 [COVID-19] (357925)

24 exp Vaccination/ (298094)

25 COVID-19 Vaccines/ (3702)

26 (immunis* or immuniz* or inoculat* or vaccin* or unvaccin*).tw,kf. (1215334)

27 or/24-26 [VACCINATION] (1259802)

28 23 and 27 [COVID-19 - VACCINATION] (33531)

29 Vaccination Refusal/ (1209)

30 ((immunis* or immuniz* or inoculat* or vaccin*) adj5 (accept* or ambivalen* or apprehensive* or concern or concerns or confiden* or nonconfiden* or non-confiden* or disinclin* or distrust* or dubious* or hesitan* or hesitat* or indecisiv* or indispos* or mistrust* or oppos* or readiness* or refus* or reject* or reluctan* or resist* or skeptic* or suspici* or tentativ* or trust* or undecided or uncertain* or unsure* or unwilling* or vacillat*)).tw,kf. (36414)

31 (antivaccine? or anti-vaccine? or antivaccinat* or anti-vaccinat*).tw,kf. (1509)

32 *Patient Acceptance of Healthcare/ (30538)

33 or/29-32 [VACCINE HESITANCY ETC] (66966)

34 28 and 33 [COVID-19 - VACCINE HESITANCY ETC] (2598)

35 exp Animals/ not Humans/ (17297244)

36 34 not 35 [ANIMAL-ONLY REMOVED] (2542)

37 36 use ppez [MEDLINE RECORDS] (1350)

38 coronavirus disease 2019/ (224324)

39 severe acute respiratory syndrome coronavirus 2/ (109504)

40 Coronavirinae/ (5056)

41 Betacoronavirus/ (40876)

42 coronavirus infection/ (57620)

43 (COVID-19 or COVID19).tw,kw. (283345)

44 ((coronavirus* or corona virus*) and (hubei or wuhan or beijing or shanghai)).tw,kw. (10269)

45 (wuhan adj5 virus*).tw,kw. (541)

46 (2019-nCoV or 19nCoV or 2019nCoV).tw,kw. (3463)

47 (nCoV or n-CoV or "CoV 2" or CoV2).tw,kw. (98925)

48 (SARS-CoV-2 or SARS-CoV2 or SARSCoV-2 or SARSCoV2 or SARS2 or SARS-2 or severe acute respiratory syndrome coronavirus 2).tw,kw. (107680)

49 (2019-novel CoV or Sars-coronavirus2 or Sars-coronavirus-2 or SARS-like coronavirus* or ((novel or new or nouveau) adj2 (CoV or nCoV or covid or coronavirus* or corona virus or Pandemi*2)) or (coronavirus* and pneumonia)).tw,kw. (38103)

50 (novel coronavirus* or novel corona virus* or novel CoV).tw,kw. (19368)

51 ((coronavirus* or corona virus*) adj2 "2019").tw,kw. (62466)

52 ((coronavirus* or corona virus*) adj2 "19").tw,kw. (9805)

53 (coronavirus 2 or corona virus 2).tw,kw. (32149)

54 (OC43 or NL63 or 229E or HKU1 or HCoV* or Sars-coronavirus*).tw,kw. (7894)

55 (coronavirus* or corona virus*).ti. (45527)

56 ("B.1.1.7" or "B.1.351" or "B.1.617" or "B.1.617.2" or "B.1.427" or "B.1.429").tw,kw. (902)

57 ("P.1" and (Brazil* or variant?)).tw,kw. (3550)

58 ((alpha or beta or delta or gamma or lambda) adj3 variant?).tw,kw. (12165)

59 or/38-58 [COVID-19] (368327)

60 vaccination/ (264865)

61 SARS-CoV-2 vaccine/ (7628)

62 (immunis* or immuniz* or inoculat* or vaccin* or unvaccin*).tw,kw. (1220388)

63 or/60-62 [VACCINATION] (1258287)

64 59 and 63 [COVID-19 - VACCINATION] (34497)

65 vaccine hesitancy/ (1241)

66 vaccination refusal/ (1209)

67 ((immunis* or immuniz* or inoculat* or vaccin*) adj5 (accept* or ambivalen* or apprehensive* or concern or concerns or confiden* or nonconfiden* or non-confiden* or disinclin* or distrust* or dubious* or hesitan* or hesitat* or indecisiv* or indispos* or mistrust* or oppos* or readiness* or refus* or reject* or reluctan* or resist* or skeptic* or suspici* or tentativ* or trust* or undecided or uncertain* or unsure* or unwilling* or vacillat*)).tw,kw. (36573)

68 (antivaccine? or anti-vaccine? or antivaccinat* or anti-vaccinat*).tw,kw. (1562)

69 *patient attitude/ (36463)

70 or/65-69 [VACCINE HESITANCY ETC] (74145)

71 64 and 70 [COVID-19 - VACCINE HESITANCY ETC] (2727)

72 exp animal/ or exp animal experimentation/ or exp animal model/ or exp animal experiment/ or nonhuman/ or exp vertebrate/ (56096555)

73 exp human/ or exp human experimentation/ or exp human experiment/ (43377661)

74 72 not 73 (12720770)

75 71 not 74 [ANIMAL-ONLY REMOVED] (2675)

76 75 use emczd [EMBASE RECORDS] (1337)

77 Coronavirus/ (14454)

78 (COVID-19 or COVID19).tw,id. (273304)

79 ((coronavirus* or corona virus*) and (hubei or wuhan or beijing or shanghai)).tw,id. (9924)

80 (wuhan adj5 virus*).tw,id. (515)

81 (2019-nCoV or 19nCoV or 2019nCoV).tw,id. (2671)

82 (nCoV or n-CoV or "CoV 2" or CoV2).tw,id. (91885)

83 (SARS-CoV-2 or SARS-CoV2 or SARSCoV-2 or SARSCoV2 or SARS2 or SARS-2 or severe acute respiratory syndrome coronavirus 2).tw,id. (93908)

84 (2019-novel CoV or Sars-coronavirus2 or Sars-coronavirus-2 or SARS-like coronavirus* or ((novel or new or nouveau) adj2 (CoV or nCoV or covid or coronavirus* or corona virus or Pandemi*2)) or (coronavirus* and pneumonia)).tw,id. (36307)

85 (novel coronavirus* or novel corona virus* or novel CoV).tw,id. (18268)

86 ((coronavirus* or corona virus*) adj2 "2019").tw,id. (61602)

87 ((coronavirus* or corona virus*) adj2 "19").tw,id. (9505)

88 (coronavirus 2 or corona virus 2).tw,id. (31198)

89 (OC43 or NL63 or 229E or HKU1 or HCoV* or Sars-coronavirus*).tw,id. (7555)

90 (coronavirus* or corona virus*).ti. (45527)

91 ("B.1.1.7" or "B.1.351" or "B.1.617" or "B.1.617.2" or "B.1.427" or "B.1.429").tw,id. (882)

92 ("P.1" and (Brazil* or variant?)).tw,id. (3528)

93 ((alpha or beta or delta or gamma or lambda) adj3 variant?).tw,id. (12108)

94 or/77-93 [COVID-19] (347434)

95 Immunization/ (172166)

96 (immunis* or immuniz* or inoculat* or vaccin* or unvaccin*).tw,id. (1209199)

97 or/95-96 [VACCINATION] (1249595)

98 94 and 97 [COVID-19 - VACCINATION] (30164)

99 ((immunis* or immuniz* or inoculat* or vaccin*) adj5 (accept* or ambivalen* or apprehensive* or concern or concerns or confiden* or nonconfiden* or non-confiden* or disinclin* or distrust* or dubious* or hesitan* or hesitat* or indecisiv* or indispos* or mistrust* or oppos* or readiness* or refus* or reject* or reluctan* or resist* or skeptic* or suspici* or tentativ* or trust* or undecided or uncertain* or unsure* or unwilling* or vacillat*)).tw,id. (36156)

100 (antivaccine? or anti-vaccine? or antivaccinat* or anti-vaccinat*).tw,id. (1462)

101 *Client Attitudes/ (13981)

102 or/99-101 [VACCINE HESITANCY ETC] (51013)

103 98 and 102 [COVID-19 - VACCINE HESITANCY ETC] (2421)

104 exp Animals/ not Humans/ (17297244)

105 103 not 104 [ANIMAL-ONLY REMOVED] (2374)

106 105 use ppez,emczd (2315)

107 105 not 106 [PSYCINFO RECORDS] (59)

108 37 or 76 or 107 [ALL DATABASES] (2746)

109 remove duplicates from 108 (1641) [TOTAL UNIQUE RECORDS]

110 109 use ppez [MEDLINE UNIQUE RECORDS] (1319)

111 109 use emczd [EMBASE UNIQUE RECORDS] (304)

112 109 not (110 or 111) [PSYCINFO UNIQUE RECORDS] (18)

***************************

CINAHL

| # | Query | Limiters/Expanders | Last Run Via | Results |
| --- | --- | --- | --- | --- |
| S31 | S26 AND S30 | Search modes - Boolean/Phrase | Interface - EBSCOhost Research Databases  Search Screen - Advanced Search  Database - CINAHL Plus with Full Text | 385 |
| S30 | S27 OR S28 OR S29 | Search modes - Boolean/Phrase | Interface - EBSCOhost Research Databases  Search Screen - Advanced Search  Database - CINAHL Plus with Full Text | 4,452 |
| S29 | TI ( antivaccine# or (anti W0 vaccine#) or antivaccinat* or (anti W0 vaccinat*) ) OR AB ( antivaccine# or (anti W0 vaccine#) or antivaccinat* or (anti W0 vaccinat*) ) | Search modes - Boolean/Phrase | Interface - EBSCOhost Research Databases  Search Screen - Advanced Search  Database - CINAHL Plus with Full Text | 262 |
| S28 | TI ( (immunis* or immuniz* or inoculat* or vaccin*) N5 (accept* or ambivalen* or apprehensive* or concern or concerns or confiden* or nonconfiden* or non-confiden* or disinclin* or distrust* or dubious* or hesitan* or hesitat* or indecisiv* or indispos* or mistrust* or oppos* or readiness* or refus* or reject* or reluctan* or resist* or skeptic* or suspici* or tentativ* or trust* or undecided or uncertain* or unsure* or unwilling* or vacillat*) ) OR AB ( (immunis* or immuniz* or inoculat* or vaccin*) N5 (accept* or ambivalen* or apprehensive* or concern or concerns or confiden* or nonconfiden* or non-confiden* or disinclin* or distrust* or dubious* or hesitan* or hesitat* or indecisiv* or indispos* or mistrust* or oppos* or readiness* or refus* or reject* or reluctan* or resist* or skeptic* or suspici* or tentativ* or trust* or undecided or uncertain* or unsure* or unwilling* or vacillat*) ) | Search modes - Boolean/Phrase | Interface - EBSCOhost Research Databases  Search Screen - Advanced Search  Database - CINAHL Plus with Full Text | 4,242 |
| S27 | (MH "Anti-Vaccination Movement") | Search modes - Boolean/Phrase | Interface - EBSCOhost Research Databases  Search Screen - Advanced Search  Database - CINAHL Plus with Full Text | 106 |
| S26 | S21 AND S25 | Search modes - Boolean/Phrase | Interface - EBSCOhost Research Databases  Search Screen - Advanced Search  Database - CINAHL Plus with Full Text | 3,824 |
| S25 | S22 OR S23 OR S24 | Search modes - Boolean/Phrase | Interface - EBSCOhost Research Databases  Search Screen - Advanced Search  Database - CINAHL Plus with Full Text | 76,124 |
| S24 | TI ( immunis* or immuniz* or inoculat* or vaccin* or unvaccin* ) OR AB ( immunis* or immuniz* or inoculat* or vaccin* or unvaccin* ) | Search modes - Boolean/Phrase | Interface - EBSCOhost Research Databases  Search Screen - Advanced Search  Database - CINAHL Plus with Full Text | 67,283 |
| S23 | (MH "COVID-19 Vaccines") | Search modes - Boolean/Phrase | Interface - EBSCOhost Research Databases  Search Screen - Advanced Search  Database - CINAHL Plus with Full Text | 1,498 |
| S22 | (MH "Immunization+") | Search modes - Boolean/Phrase | Interface - EBSCOhost Research Databases  Search Screen - Advanced Search  Database - CINAHL Plus with Full Text | 29,498 |
| S21 | S1 OR S2 OR S3 OR S4 OR S5 OR S6 OR S7 OR S8 OR S9 OR S10 OR S11 OR S12 OR S13 OR S14 OR S15 OR S16 OR S17 OR S18 OR S19 OR S20 | Search modes - Boolean/Phrase | Interface - EBSCOhost Research Databases  Search Screen - Advanced Search  Database - CINAHL Plus with Full Text | 57,821 |
| S20 | TI ( (alpha or beta or delta or gamma or lambda) N3 variant# ) OR AB ( (alpha or beta or delta or gamma or lambda) N3 variant# ) | Search modes - Boolean/Phrase | Interface - EBSCOhost Research Databases  Search Screen - Advanced Search  Database - CINAHL Plus with Full Text | 223 |
| S19 | TI ( "P.1" and (Brazil* or variant#) ) OR AB ( "P.1" and (Brazil* or variant#) ) | Search modes - Boolean/Phrase | Interface - EBSCOhost Research Databases  Search Screen - Advanced Search  Database - CINAHL Plus with Full Text | 11 |
| S18 | TI ( "B.1.1.7" or "B.1.351" or "B.1.617" or "B.1.617.2" or "B.1.427" or "B.1.429" ) OR AB ( "B.1.1.7" or "B.1.351" or "B.1.617" or "B.1.617.2" or "B.1.427" or "B.1.429" ) | Search modes - Boolean/Phrase | Interface - EBSCOhost Research Databases  Search Screen - Advanced Search  Database - CINAHL Plus with Full Text | 55 |
| S17 | TI coronavirus* or "corona virus" or "corona viruses" | Search modes - Boolean/Phrase | Interface - EBSCOhost Research Databases  Search Screen - Advanced Search  Database - CINAHL Plus with Full Text | 5,834 |
| S16 | TI ( OC43 or NL63 or 229E or HKU1 or HCoV* or "Sars-coronavirus" or "Sars-coronaviruses" ) OR AB ( OC43 or NL63 or 229E or HKU1 or HCoV* or "Sars-coronavirus" or "Sars-coronaviruses" ) | Search modes - Boolean/Phrase | Interface - EBSCOhost Research Databases  Search Screen - Advanced Search  Database - CINAHL Plus with Full Text | 269 |
| S15 | TI ( "coronavirus 2" or "corona virus 2" ) OR AB ( "coronavirus 2" or "corona virus 2" ) | Search modes - Boolean/Phrase | Interface - EBSCOhost Research Databases  Search Screen - Advanced Search  Database - CINAHL Plus with Full Text | 2,851 |
| S14 | TI ( (coronavirus* or "corona virus" or "corona viruses") N2 "19" ) OR AB ( (coronavirus* or "corona virus" or "corona viruses") N2 "19" ) | Search modes - Boolean/Phrase | Interface - EBSCOhost Research Databases  Search Screen - Advanced Search  Database - CINAHL Plus with Full Text | 172 |
| S13 | TI ( (coronavirus* or "corona virus" or "corona viruses") N2 "2019" ) OR AB ( (coronavirus* or "corona virus" or "corona viruses") N2 "2019" ) | Search modes - Boolean/Phrase | Interface - EBSCOhost Research Databases  Search Screen - Advanced Search  Database - CINAHL Plus with Full Text | 8,703 |
| S12 | TI ( "novel coronavirus" or "novel coronaviruses" or "novel corona virus" or "novel corona viruses" or "novel CoV" ) OR AB ( "novel coronavirus" or "novel coronaviruses" or "novel corona virus" or "novel corona viruses" or "novel CoV" ) | Search modes - Boolean/Phrase | Interface - EBSCOhost Research Databases  Search Screen - Advanced Search  Database - CINAHL Plus with Full Text | 2,247 |
| S11 | TI ( ("2019-novel CoV" or "Sars-coronavirus2" or "Sars-coronavirus-2" or "SARS-like coronavirus" or "SARS-like coronaviruses" or ((novel or new or nouveau) N2 (CoV or nCoV or covid or coronavirus* or "corona virus" or pandemi*)) or (coronavirus* and pneumonia)) ) OR AB ( ("2019-novel CoV" or "Sars-coronavirus2" or "Sars-coronavirus-2" or "SARS-like coronavirus" or "SARS-like coronaviruses" or ((novel or new or nouveau) N2 (CoV or nCoV or covid or coronavirus* or "corona virus" or pandemi*)) or (coronavirus* and pneumonia)) ) | Search modes - Boolean/Phrase | Interface - EBSCOhost Research Databases  Search Screen - Advanced Search  Database - CINAHL Plus with Full Text | 4,554 |
| S10 | TI ( "SARS-CoV-2" or "SARS-CoV2" or "SARSCoV-2" or SARSCoV2 or SARS2 or "SARS-2" or "severe acute respiratory syndrome coronavirus 2" ) OR AB ( "SARS-CoV-2" or "SARS-CoV2" or "SARSCoV-2" or SARSCoV2 or SARS2 or "SARS-2" or "severe acute respiratory syndrome coronavirus 2" ) | Search modes - Boolean/Phrase | Interface - EBSCOhost Research Databases  Search Screen - Advanced Search  Database - CINAHL Plus with Full Text | 8,507 |
| S9 | TI ( nCoV or n-CoV or "CoV 2" or CoV2 ) OR AB ( nCoV or n-CoV or "CoV 2" or CoV2 ) | Search modes - Boolean/Phrase | Interface - EBSCOhost Research Databases  Search Screen - Advanced Search  Database - CINAHL Plus with Full Text | 152 |
| S8 | TI ( "2019-nCoV" or 19nCoV or 2019nCoV ) OR AB ( "2019-nCoV" or 19nCoV or 2019nCoV ) | Search modes - Boolean/Phrase | Interface - EBSCOhost Research Databases  Search Screen - Advanced Search  Database - CINAHL Plus with Full Text | 262 |
| S7 | TI wuhan N5 virus* OR AB wuhan N5 virus* | Search modes - Boolean/Phrase | Interface - EBSCOhost Research Databases  Search Screen - Advanced Search  Database - CINAHL Plus with Full Text | 52 |
| S6 | TI ( ((coronavirus* or (corona W0 virus*)) and (hubei or wuhan or beijing or shanghai)) ) OR AB ( ((coronavirus* or (corona W0 virus*)) and (hubei or wuhan or beijing or shanghai)) ) | Search modes - Boolean/Phrase | Interface - EBSCOhost Research Databases  Search Screen - Advanced Search  Database - CINAHL Plus with Full Text | 1,026 |
| S5 | TI ( ("COVID-19" or COVID19) ) OR AB ( ("COVID-19" or COVID19) ) | Search modes - Boolean/Phrase | Interface - EBSCOhost Research Databases  Search Screen - Advanced Search  Database - CINAHL Plus with Full Text | 47,720 |
| S4 | (MH "Coronavirus Infections") | Search modes - Boolean/Phrase | Interface - EBSCOhost Research Databases  Search Screen - Advanced Search  Database - CINAHL Plus with Full Text | 9,927 |
| S3 | (MH "Coronavirus") | Search modes - Boolean/Phrase | Interface - EBSCOhost Research Databases  Search Screen - Advanced Search  Database - CINAHL Plus with Full Text | 989 |
| S2 | (MH "SARS-CoV-2") | Search modes - Boolean/Phrase | Interface - EBSCOhost Research Databases  Search Screen - Advanced Search  Database - CINAHL Plus with Full Text | 421 |
| S1 | (MH "COVID-19") | Search modes - Boolean/Phrase | Interface - EBSCOhost Research Databases  Search Screen - Advanced Search  Database - CINAHL Plus with Full Text | 17,373 |

Web of Science

12

(#8) AND #11

[567](https://www-webofscience-com.myaccess.library.utoronto.ca/wos/woscc/summary/1b590616-ec1f-4459-9647-2aeacfc37dbf-01b4ab5f/relevance/1)

11

(#9) OR #10

[20,550](https://www-webofscience-com.myaccess.library.utoronto.ca/wos/woscc/summary/3164c78b-2078-44bb-8db0-62ea1b319879-01b49e74/relevance/1)

10

antivaccine* or (anti NEAR/0 vaccine*) or antivaccinat* or (anti NEAR/0 vaccinat*) (Topic)

[1,143](https://www-webofscience-com.myaccess.library.utoronto.ca/wos/woscc/summary/0eab6c40-11c3-455c-a294-11c043ca3f0c-01b49699/relevance/1)

9

(immunis* or immuniz* or inoculat* or vaccin*) NEAR/5 (accept* or ambivalen* or apprehensive* or concern or concerns or confiden* or nonconfiden* or non-confiden* or disinclin* or distrust* or dubious* or hesitan* or hesitat* or indecisiv* or indispos* or mistrust* or oppos* or readiness* or refus* or reject* or reluctan* or resist* or skeptic* or suspici* or tentativ* or trust* or undecided or uncertain* or unsure* or unwilling* or vacillat*) (Topic)

[19,757](https://www-webofscience-com.myaccess.library.utoronto.ca/wos/woscc/summary/4e37cd7d-746e-40c9-91c3-fd6ddad68869-01b48e43/relevance/1)

8

(#6) AND #7

[9,786](https://www-webofscience-com.myaccess.library.utoronto.ca/wos/woscc/summary/3cbef67e-5c81-47ee-8789-c071255daefa-01b484ed/relevance/1)

7

immunis* or immuniz* or inoculat* or vaccin* or unvaccin* (Topic)

[602,581](https://www-webofscience-com.myaccess.library.utoronto.ca/wos/woscc/summary/90f2f96d-1f5c-4738-9870-c3d51d41fbe8-01b47fa0/relevance/1)

6

((((#1) OR #2) OR #3) OR #4) OR #5

[101,470](https://www-webofscience-com.myaccess.library.utoronto.ca/wos/woscc/summary/8a56e367-1485-45f7-b662-0931db3dd5e8-01b47942/relevance/1)

5

"B.1.1.7" or "B.1.351" or "B.1.617" or "B.1.617.2" or "B.1.427" or "B.1.429" (Topic) or "P.1" and (Brazil* or variant*) (Topic) or (alpha or beta or delta or gamma or lambda) NEAR/3 variant* (Topic)

[16,608](https://www-webofscience-com.myaccess.library.utoronto.ca/wos/woscc/summary/cd42413c-74a8-4261-a8ac-e294d3591bed-01b46e86/relevance/1)

4

"novel coronavirus" or "novel coronaviruses" or "novel corona virus" or "novel corona viruses" or "novel CoV" (Topic) or (coronavirus* or "corona virus" or "corona viruses") NEAR/2 "2019" (Topic) or (coronavirus* or "corona virus" or "corona viruses") NEAR/2 "19" (Topic) or "coronavirus 2" or "corona virus 2" (Topic) or OC43 or NL63 or 229E or HKU1 or HCoV* or "Sars-coronavirus" or "Sars-corona-virus" (Topic) or coronavirus* or "corona virus" or "corona viruses" (Title)

[57,078](https://www-webofscience-com.myaccess.library.utoronto.ca/wos/woscc/summary/ff3604cc-1b29-43d6-947f-4e7c62bde983-01b46669/relevance/1)

3

"2019-nCoV" or 19nCoV or 2019nCoV (Topic) or nCoV or "n-CoV" or "CoV 2" or CoV2 (Topic) or "SARS-CoV-2" or "SARS-CoV2" or "SARSCoV-2" or SARSCoV2 or SARS2 or "SARS-2" or "severe acute respiratory syndrome coronavirus 2" (Topic) or "2019-novel CoV" or "Sars-coronavirus2" or "Sars-coronavirus-2" or "SARS-like coronavirus" or "SARS-like coronaviruses" (Topic) or (novel or new or nouveau) NEAR/2 (CoV or nCoV or covid or coronavirus* or "corona virus" or pandemi*) (Topic) or coronavirus* and pneumonia (Topic)

[59,619](https://www-webofscience-com.myaccess.library.utoronto.ca/wos/woscc/summary/9e989f91-77a5-4f3d-8bd7-582a1f40fa3e-01b42f26/relevance/1)

2

wuhan NEAR/5 virus* (Title) or wuhan NEAR/5 virus* (Abstract) or wuhan NEAR/5 virus* (Author Keywords)

[301](https://www-webofscience-com.myaccess.library.utoronto.ca/wos/woscc/summary/bb33da6b-52c3-4775-b8eb-f1819dc9da02-01b40b0e/relevance/1)

1

TS=((coronavirus* or "corona virus" or "corona viruses") and (hubei or wuhan or beijing or shanghai))

[6,264](https://www-webofscience-com.myaccess.library.utoronto.ca/wos/woscc/summary/975dea52-8a7a-4d6a-832b-9e7282830539-01b4398c/relevance/1)

## eFile 1b. Grey Literature Search Strategy

Cochrane

<https://covid-19.cochrane.org/>

Vaccine + confidence

Vaccines + confidence

Vaccination + confidence

Vaccinations + confidence

Vaccine + hesitancy

Vaccines + hesitancy

Vaccination + hesitancy

Vaccinations + hesitancy

Vaccine + nonconfidence

Vaccines + nonconfidence

Vaccination + nonconfidence

Vaccinations + nonconfidence

Vaccine + non-confidence

Vaccines + non-confidence

Vaccination + non-confidence

Vaccinations + non-confidence

Vaccine + refuse

Vaccines + refuse

Vaccination + refuse

Vaccinations + refuse

Vaccine + refuses

Vaccines + refuses

Vaccination + refuses

Vaccinations + refuses

Vaccine + refused

Vaccines + refused

Vaccination + refused

Vaccinations + refused

Vaccine + refusing

Vaccines + refusing

Vaccination + refusing

Vaccinations + refusing

Vaccine + refusal

Vaccines + refusal

Vaccination + refusal

Vaccinations + refusal

Vaccine + reluctant

Vaccines + reluctant

Vaccination + reluctant

Vaccinations + reluctant

Vaccine + reluctance

Vaccines + reluctance

Vaccination + reluctance

Vaccinations + reluctance

Vaccine + skeptic

Vaccines + skeptic

Vaccination + skeptic

Vaccinations + skeptic

Vaccine + skeptical

Vaccines + skeptical

Vaccination + skeptical

Vaccinations + skeptical

Vaccine + skepticism

Vaccines + skepticism

Vaccination + skepticism

Vaccinations + skepticism

Vaccine + distrust

Vaccines + distrust

Vaccination + distrust

Vaccinations + distrust

Vaccine + distrusted

Vaccines + distrusted

Vaccination + distrusted

Vaccinations + distrusted

Vaccine + distrusting

Vaccines + distrusting

Vaccination + distrusting

Vaccinations + distrusting

Vaccine + Trust

Vaccines + Trust

Vaccination + Trust

Vaccinations + Trust

Vaccine + Trusts

Vaccines + Trusts

Vaccination + Trusts

Vaccinations + Trusts

Vaccine + Trusted

Vaccines + Trusted

Vaccination + Trusted

Vaccinations + Trusted

Vaccine + Trusting

Vaccines + Trusting

Vaccination + Trusting

Vaccinations + Trusting

Covid-END

<https://www.mcmasterforum.org/networks/covid-end/resources-to-support-decision-makers/Inventory-of-best-evidence-syntheses>

Scanned public health measures, economic & social responses, health system arrangements, clinical managment

*Results found*

<https://www.mcmasterforum.org/find-evidence/covid-19-evidence/covid-19-evidence-from-hse-and-sse>

*No results*

L-OVE

<https://app.iloveevidence.com/>

(vaccine OR vaccines OR vaccinat*) AND (Hesitancy OR Confidence OR Nonconfidence OR Non-confidence OR Refuse* OR Refusal* OR Reluctan* OR Skeptic* OR Distrust* OR Trust*)

*3864 records (no option to limit by date)*

LTC Covid

<https://ltccovid.org/>

<https://ltccovid.org/resources/>

Vaccination + Staff

*Results found (picked selectively from list)*

<https://ltccovid.org/completed-or-ongoing-research-projects-on-covid-19-and-long-term-care/>

*Nothing relevant*

UNCOVER

<https://www.ed.ac.uk/usher/uncover/register-of-reviews>

(*Use CTRL+F to browse full list*)

*Nothing unique*

ClinicalTrials.gov – Covid-19 resources

<https://clinicaltrials.gov/ct2/results?cond=COVID-19>

34 Studies found for: Hesitancy OR Confidence OR Nonconfidence OR Non-Confidence OR Refuse OR Refuses OR Refused OR Refusal OR Reluctant OR Reluctance OR Skeptic OR Skeptical OR Skepticism OR Distrust OR Trust | COVID-19 | vaccination | First posted from 08/12/2021 to 02/15/2022

WHO Covid-19 Database

<https://search.bvsalud.org/global-literature-on-novel-coronavirus-2019-ncov/>

(tw:(COVID or "COVID-19" or COVID19 or coronavirus or coronaviruses or "corona virus" or "corona viruses" )) AND (tw:(vaccine or vaccines or vaccination or vaccinations or vaccinate or vaccinated or vaccinates or vaccinating)) AND (tw:(Hesitancy OR Confidence OR Nonconfidence OR Non-Confidence OR Refuse OR Refuses OR Refused OR Refusal OR Reluctant OR Reluctance OR Skeptic OR Skeptical OR Skepticism OR Distrust OR Trust)) + Main Subject: COVID-19, COVID-19 vaccines, Vaccination Refusal, Health Personnel + Year 2021 or 2022 – 1154 records

Epistemonikos

<https://www.epistemonikos.cl/living-evidence/>

*Covered under COVID-19 L-OVE*

TRIP

<https://www.tripdatabase.com>

("covid-19" OR covid19 OR covid OR coronavirus* OR "corona virus" OR "corona viruses") AND (vaccine OR vaccines OR vaccination OR vaccinations OR vaccinate OR vaccinated OR vaccinates OR vaccinating) AND (hesitancy OR confidence OR nonconfidence OR non-confidence OR refuse OR refuses OR refused OR refusal OR reluctant OR reluctance OR skeptic OR skeptical OR skepticism OR distrust OR trust) from_date:2021 – 123 results

*Selected potentially relevant items from results*

## eFile 2. Data Charting Tool

| **Publication Details** | | | | | | |
| --- | --- | --- | --- | --- | --- | --- |
| Author(s) | Article Title | Source (Journal) Title | Country | Publication Type | Date of Publication | Date of Study |

| **Education Description** | | | | | | | | |
| --- | --- | --- | --- | --- | --- | --- | --- | --- |
| Education Initiative Title | Education Setting | Objective | Key Education Content  (what was the key material/information shared?) | Delivery Format  (e.g. virtual or in person) | Delivery Structure  (e.g. group, one-on-one) | Education Facilitators | Education Audience | Delivery Timeline  (e.g. duration and frequency) |

## eTable 1. Delivery Format

| **Delivery Format** | **Round 1 (n=13)** | | | **Round 2 (n=19)** | | |
| --- | --- | --- | --- | --- | --- | --- |
|  | n | (%) | References | n | % | References |
| **Group-Based** | 12 | (92) |  | 15 | (79) |  |
| Presentation | 11 | (85) |  | 13 | (68) |  |
| Virtual | 7 | (64) | (4–10) | 9 | (69) | (11–19) |
| In-Person | 2 | (18) | (20,21) | 1 | (8) | (22) |
| Combination | 1 | (9) | (23) | 1 | (8) | (24) |
| Unclear | 1 | (9) | (25) | 2 | (15) | (26,27) |
| Community Events | 1 | (8) |  | 3 | (16) |  |
| In-Person | 1 | (100) | (28) | 3 | (100) | (12,29,30) |
| Small group discussions | 1 | (8) |  | 2 | (11) |  |
| In-Person | 1 | (100) | (8) | 2 | (100) | (12,19) |
| **Individual-Based** | 5 | (38) |  | 10 | (53) |  |
| Phone Call (virtual) | 2 | (15) | (22,23) | 5 | (26) | (12,27,31–33) |
| Hotlines | 0 |  | N/A | 2 | (40) | (12,33) |
| In-person, physician visit | 1 | (8) | (20) | 2 | (11) | (26,34) |
| In-person, on-site consultation/conversation/counselling | 3 | (23) | (8,20,25) | 2 | (11) | (29,32) |
| Door-to-door communication | 0 |  | N/A | 2 | (11) | (15,29) |

Note: categories were not mutually exclusive.

| Number of Interventions Introduced within a Study | **Round 1 (n=13)** | | | **Round 2 (n=19)** | | |
| --- | --- | --- | --- | --- | --- | --- |
|  | n | (%) | References | n | (%) | References |
| 1 | 9 | (69) |  | 12 | (63) |  |
| 2 | 2 | (15) | (25,28) | 5 | (26) | (15,26,27,32,33) |
| 3 | 2 | (15) | (8,20) | 1 | (5) | (29) |
| 4 | 0 |  |  | 1 | (5) | (12) |

## eTable 2. Number of Interventions

## eTable 3. Target Population

| Target Population | **Round 1 (n=13)** | | | **Round 2 (n=19)** | | |
| --- | --- | --- | --- | --- | --- | --- |
|  | n | (%) | References | n | (%) | References |
| **Minority and Marginalized Communities** | 4 | (31) |  | 12 | (63) |  |
| Diverse Racial and Ethnic Groups | 3 | (75) | (9,10,35) | 4 | (33) | (12,13,15,30) |
| Immigrants | 0 |  |  | 6 | (50) | (17–19,29,32,33) |
| Hispanic and Latinx Communities | 1 | (25) | (5) | 1 | (8) | (29) |
| African Americans | 2 | (50) | (5,10) | 2 | (17) | (16,22) |
| **Other Localized Communities** | 6 | (46) |  | 7 | (37) |  |
| Patient Group | 3 | (50) | (4,28,35) | 4 | (57) | (26,27,31,34) |
| Pharmacy Network | 2 | (33) | (7,23) | 0 |  | N/A |
| Soldiers | 1 | (17) | (20) | 1 | (14) | (24) |
| High-risk occupations | 0 |  | N/A | 1 | (14) | (12) |
| General Public | 0 |  | N/A | 1 | (14) | (11) |
| **Healthcare and Long-Term Care Sectors** | 5 | (38) |  | 4 | (21) |  |
| LTC Home Staff | 2 | (40) | (4,6) | 0 |  | N/A |
| Hospital Staff | 2 | (40) | (21,25) | 0 |  | N/A |
| Community Practitioners | 1 | (20) | (9) | 3 | (75) | (11,12,14) |
| Veterans Affairs Members | 0 |  | N/A | 1 | (25) | (27) |

Note: categories were not mutually exclusive.

## eTable 4. Facilitator Type

| Facilitator Type | **Round 1 (n=13)** | | | **Round 2 (n=19)** | | |
| --- | --- | --- | --- | --- | --- | --- |
|  | n | (%) | References | n | (%) | References |
| Healthcare Professional | 13 | (100) | (4–10,20,21,23,25,28,35) | 16 | (84) | (11,12,14,15,17–19,22,24,26,27,29–31,33,34) |
| Faith Leader | 2 | (15) | (5,28) | 2 | (11) | (12,32) |
| Community Leader | 2 | (15) | (4,28) | 8 | (42) | (12,13,15,17,19,22,29,33) |
| Academic Leader | 2 | (15) | (5,6) | 3 | (16) | (11,13,14) |

Note: categories were not mutually exclusive.

## eTable 5. Number of Facilitators

| Number of Facilitators | **Round 1 (n=13)** | | | **Round 2 (n=19)** | | |
| --- | --- | --- | --- | --- | --- | --- |
|  | n | (%) | References | n | (%) | References |
| 1 | 8 | (62) | (7,9,10,20,21,23,25,35) | 10 | (53) | (13,16,18,24,26,27,30–32,34) |
| 2 | 3 | (23) | (4,6,8) | 6 | (32) | (11,14,17,19,22,33) |
| 3 | 1 | (8) | (28) | 3 | (16) | (12,15,29) |
| 4 | 1 | (8) | (5) | 0 | (0) |  |

References

1. Presseau J, Desveaux L, Allen U, et al. Behavioural science principles for supporting COVID-19 vaccine confidence and uptake among Ontario health care workers. Sci Briefs Ont COVID-19 Sci Advis Table. 2021;2(12).

2. Kahale LA, Elkhoury R, El Mikati I, Pardo-Hernandez H, Khamis AM, Schünemann HJ, et al. Tailored PRISMA 2020 flow diagrams for living systematic reviews: a methodological survey and a proposal. F1000Research. 2022 Jan 28;10:192.

3. Page MJ, McKenzie JE, Bossuyt PM, Boutron I, Hoffmann TC, Mulrow CD, et al. The PRISMA 2020 statement: an updated guideline for reporting systematic reviews. BMJ. 2021 Mar 29;372:n71.

4. Kelkar AH, Blake JA, Cherabuddi K, Cornett H, McKee BL, Cogle CR. Vaccine Enthusiasm and Hesitancy in Cancer Patients and the Impact of a Webinar. Healthcare. 2021 Mar 19;9(3):351.

5. Peteet B, Belliard JC, Abdul-Mutakabbir J, Casey S, Simmons K. Community-academic partnerships to reduce COVID-19 vaccine hesitancy in minoritized communities. eClinicalMedicine [Internet]. 2021 Apr 1 [cited 2022 Mar 1];34. Available from: https://www.thelancet.com/journals/eclinm/article/PIIS2589-5370(21)00114-0/fulltext

6. Berry SD, Johnson KS, Myles L, Herndon L, Montoya A, Fashaw S, et al. Lessons learned from frontline skilled nursing facility staff regarding COVID‐19 vaccine hesitancy. J Am Geriatr Soc. 2021 May;69(5):1140–6.

7. Traynor K. Pharmacists promote COVID-19 vaccine acceptance in rural locales. Am J Health Syst Pharm. 2021 Aug 1;78(15):1361–2.

8. Feifer RA, Bethea L, White EM. Racial Disparities in COVID-19 Vaccine Acceptance: Building Trust to Protect Nursing Home Staff and Residents. J Am Med Dir Assoc. 2021 Sep;22(9):1853-1855.e1.

9. Quinn SC, Andrasik MP. Addressing Vaccine Hesitancy in BIPOC Communities — Toward Trustworthiness, Partnership, and Reciprocity. N Engl J Med. 2021 Jul 8;385(2):97–100.

10. NICE. COVID-19 vaccine hesitancy – debunking the myths using a community engagement approach underpinned by NICE guidance [Internet]. National Institute for Health and Care Excellence. 2021. Available from: https://www.nice.org.uk/sharedlearning/covid-19-vaccine-hesitancy-debunking-the-myths-using-a-community-engagement-approach-underpinned-by-nice-guidance

11. Abdel-Qader DH, Hayajneh W, Albassam A, Obeidat NM, Belbeisi AM, Al Mazrouei N, et al. Pharmacists-physicians collaborative intervention to reduce vaccine hesitancy and resistance: A randomized controlled trial. Vaccine X. 2022 Apr;10:100135.

12. AuYoung M, Rodriguez Espinosa P, Chen WT, Juturu P, Young MEDT, Casillas A, et al. Addressing racial/ethnic inequities in vaccine hesitancy and uptake: lessons learned from the California alliance against COVID-19. J Behav Med. 2022 Jan 22;1–14.

13. Wagner EF, Langwerden RJ, Morris SL, Ward MK, Trepka MJ, Campa AL, et al. Virtual town halls addressing vaccine hesitancy among racial and ethnic minorities: Preliminary findings. J Am Pharm Assoc. 2022 Jan 1;62(1):317–25.

14. Katzman JG, Thornton K, Sosa N, Tomedi L, Hayes L, Sievers M, et al. Educating health professionals about COVID-19 with ECHO telementoring. Am J Infect Control. 2022 Mar;50(3):283–8.

15. Ginder-Vogel K. Combating Racial Disparities in COVID-19 Vaccination [Internet]. School of Pharmacy. 2021 [cited 2022 Dec 1]. Available from: https://pharmacy.wisc.edu/combating-racial-disparities-in-covid-19-vaccination/

16. Hopper L. COVID town hall takes on vaccine concerns in the Black community [Internet]. USC News. 2021 [cited 2022 Dec 1]. Available from: https://news.usc.edu/182502/usc-covid-town-hall-vaccine-concerns-black-community/

17. Bouchard K. Outreach efforts target vaccine access and reluctance in Maine’s immigrant population - Portland Press Herald [Internet]. The Portland Press Herald. 2021 [cited 2022 Dec 1]. Available from: https://www.pressherald.com/2021/03/28/outreach-efforts-target-vaccine-access-and-reluctance-in-maines-immigrant-population/

18. Tesfaye E. African Immigrant Health Groups Battle Trans-Atlantic Tide Of Vaccine Disinformation [Internet]. Georgia Public Broadcasting. [cited 2022 Dec 1]. Available from: https://www.gpb.org/news/2021/04/06/african-immigrant-health-groups-battle-trans-atlantic-tide-of-vaccine

19. Garcia S. Advocates working to get COVID-19 vaccine to Baltimore’s hard-hit Latino community [Internet]. 2021 [cited 2022 Dec 1]. Available from: https://www.baltimoresun.com/coronavirus/bs-hs-vaccine-latino-outreach-20210222-lju36ekrpzh5xoisp7j46nztya-story.html

20. Talmy T, Cohen B, Nitzan I, Ben Michael Y. Primary Care Interventions to Address COVID-19 Vaccine Hesitancy Among Israel Defense Forces Soldiers. J Community Health. 2021;46(6):1155–60.

21. Gakuba C, Sar A, Gaborieau I, Hanouz JL, Verger P. Willingness to get a COVID-19 vaccine among critical care non-medical healthcare workers and impact of a vaccine information session. Anaesth Crit Care Pain Med. 2021 Jun;40(3):100860.

22. WCAX. Vaccine outreach ramps up to reach Vt. immigrant communities [Internet]. https://www.wcax.com. 2021 [cited 2022 Dec 1]. Available from: https://www.wcax.com/2021/02/05/outreach-continuing-in-many-languages-to-educate-on-the-vaccine/

23. Abdel-Qader DH, Al Meslamani AZ, Al Mazrouei N, El-Shara AA, El Sharu H, Merghani Ali E, et al. Virtual Coaching Delivered by Pharmacists to Prevent COVID-19 Transmission. Hosp Pharm. 2021 Jul 10;00185787211032354.

24. Li PC, Theis SR, Kelly D, Ocampo T, Berglund A, Morgan D, et al. Impact of an Education Intervention on COVID-19 Vaccine Hesitancy in a Military Base Population. Mil Med. 2022 Oct 29;187(11–12):e1449–55.

25. Takamatsu A, Honda H, Kojima T, Murata K, Babcock HM. Promoting coronavirus disease 2019 (COVID-19) vaccination among healthcare personnel: A multifaceted intervention at a tertiary-care center in Japan. Infect Control Hosp Epidemiol. 2021;1–6.

26. Abou Leila R, Salamah M, El-Nigoumi S. Reducing COVID-19 Vaccine Hesitancy by Implementing Organizational Intervention in a Primary Care Setting in Bahrain. Cureus. 2021 Nov;13(11):e19282.

27. Spelman JF, Kravetz JD, Bastian L, Ruser C. Addressing COVID-19 Vaccine Acceptance Within a Large Healthcare System: a Population Health Model. J Gen Intern Med. 2022 Mar 1;37(4):954–7.

28. Moberly T. Covid-19: Vaccine hesitancy fell after vaccination programme started. BMJ. 2021 Mar 26;372:n837.

29. Marquez C, Kerkhoff AD, Naso J, Contreras MG, Castellanos Diaz E, Rojas S, et al. A multi-component, community-based strategy to facilitate COVID-19 vaccine uptake among Latinx populations: From theory to practice. PloS One. 2021;16(9):e0257111.

30. Scott T, Gutschow B, Ragavan MI, Ho K, Massart M, Ripper L, et al. A Community Partnered Approach to Promoting COVID-19 Vaccine Equity. Health Promot Pract. 2021 Nov;22(6):758–60.

31. Serper M, Liu CH, Blumberg EA, Burdzy AE, Veasey S, Halpern S, et al. A pragmatic outreach pilot to understand and overcome barriers to COVID-19 vaccination in abdominal organ transplant. Transpl Infect Dis Off J Transplant Soc. 2021 Oct;23(5):e13722.

32. Rosario I. How volunteers help Story County immigrants get COVID-19 vaccinations. Ames Tribune [Internet]. 2021 May 17 [cited 2022 Dec 1]; Available from: https://www.amestrib.com/story/news/2021/05/17/covid-19-vaccine-story-county-ames-coronavirus-how-volunteers-help-immigrants-vaccination/5035247001/

33. Wiley M. Advocates Work to Combat Vaccine Distrust in ICE Detention Facilities | KQED [Internet]. KQED. 2021 [cited 2022 Dec 1]. Available from: https://www.kqed.org/news/11869046/advocates-work-to-combat-vaccine-distrust-in-ice-detention-facilities

34. Hirshberg JS, Huysman BC, Oakes MC, Cater EB, Odibo AO, Raghuraman N, et al. Offering onsite COVID-19 vaccination to high-risk obstetrical patients: initial findings. Am J Obstet Gynecol MFM. 2021 Nov;3(6):100478.

35. NHS England. GP determined to put in personal call to every at-risk patient yet to take up COVID-19 jab offer [Internet]. News. 2021 [cited 2022 Mar 1]. Available from: https://www.england.nhs.uk/2021/02/gp-determined-to-put-in-personal-call-to-every-at-risk-patient-yet-to-take-up-covid-19-jab-offer/
